# *Amphistegina lessonii* and *Amphistegina lobifera* shell microstructure, texture and twinning pattern reflect resilience to cadmium and lead

**DOI:** 10.1038/s41598-025-94811-7

**Published:** 2025-04-26

**Authors:** A. Sancho Vaquer, E. Griesshaber, X. Yin, M. Siccha, N. Ben-Eliahu, B. Herut, E. Rahav, S. Abramovich, M. Kucera, W. W. Schmahl

**Affiliations:** 1 Department of Earth and Environmental Sciences, LMU Munich, Munich, Germany; 2 Bruker Nano Surfaces and Metrology Division, Minhang District, Shanghai, China; 3 MARUM – Centre for Marine Environmental Sciences, University of Bremen, Bremen, Germany; 4 Department of Earth and Environmental Science, Ben-Gurion University of the Negev, Beer Sheva, Israel; 5 National Institute of Oceanography, Israel Oceanographic and Limnological Research, Haifa, Israel

**Keywords:** Electron microscopy, Environmental impact, Mineralogy, Marine biology

## Abstract

Biologically secreted carbonates are archives of environmental conditions, as shell and skeletal element minerals record chemical and physical signals of the ambient environment. We report the impact of Cd^2+^ and Pb^2+^ on foraminiferal shell crystal structural and organizational characteristics, such as microstructure, texture, crystal co-orientation strength and crystal twin formation for the rotaliid foraminifera *Amphistegina lessonii* and *Amphistegina lobifera.* The investigated species lived first in Cd^2+^- and Pb^2+^-free and, at a later growth stage, in Cd^2+^- and Pb^2+^-containing water. Enrichment in Cd^2+^ was increased 4 times relative to the ecological criteria maximum concentration (CMC) for both species. For Pb^2+^, it was increased 5 times for *A. lobifera* and 6 times for *A. lessonii*. Crystal organization was measured with Electron-Backscattered-Diffraction (EBSD), shell structure was imaged with FE-SEM. We detect that the Cd^2+^ and Pb^2+^ concentrations influence the degree of shell calcite twin formation. For *A. lessonii* the addition of Cd^2+^ to the water prevents crystal twin generation, Pb^2+^ induces decreased twinned calcite secretion. For *A. lobifera*, both Cd^2+^ and Pb^2+^ significantly decrease crystal twin formation. Our study indicates that crystal twin generation by Rotaliida can be developed as a structural indicator for environmental pollution with heavy toxic elements.

**Supplementary Information:**

The online version contains supplementary material available at 10.1038/s41598-025-94811-7.

## Introduction

Microdiffraction methods such as electron backscatter diffraction in SEM and TEM (EBSD) record the orientation of all crystallographic axes of crystals. This enables the determination of crystal orientation and, for a particular sample volume, the determination of the microstructure and texture (crystallographic preferred orientation) of a crystallized material^1^. In addition, EBSD measurements provide further information on the material’s structural properties, including crystal co-orientation strength, crystal size, morphology and interlinkage, crystal twin formation, and information on the prevailing twin law. Studies that investigated the microstructure and crystallographic texture of benthic and planktonic rotaliid and benthic robertinid foraminifera shells with EBSD^2–6^ examined the following species: *Globigerinita glutinata* (Egger, 1893), *Candeina nitida* (d’Orbigny, 1839), *Ammonia tepida* (Cushman, 1926), *Amphistegina lobifera* (Larsen, 1976), *Amphistegina lessonii* (d’Orbigny, 1830), *Pulleniatina obliquiloculata* (Parker and Jones, 1865), *Globigerinoides sacculifer* (Brady, 1877), *Orbulina universa* (d’Orbigny, 1839), *Ammonia convexa* (Collins, 1958), *Operculina ammonoides* (Gronovius, 1781) and *Hoeglundina elegans* (d’Orbigny, 1826) and demonstrated that almost all investigated foraminifera exhibited one prominent material characteristic: the twinned nature of foraminiferal calcite in Rotaliida and foraminiferal aragonite in Robertina. Out of 11 modern species, only the calcite of the planktonic, clade 3 species, *G. glutinata* was not twinned.

Crystal twinning is an important material characteristic and occurs in non-biological as well as in biological structural materials. For non-biological materials, it has been shown that the generation of twins is a material property-enhancing mechanism, particularly ductility^7–10^. In the biological realm, crystal twin formation is often reported for biological aragonite, for the aragonite of bivalves, gastropods, and corals^11–17^. However, except for rotaliid shells, crystal twin formation is not typical for biological calcites. Mollusc and brachiopod calcites are not twinned. Hence, this characteristic of rotaliid and robertinid carbonate is striking and an intrinsic material property. This poses the question of whether the crystal twin signal can be used as a tool for tracing ambient perturbance, such as environmental aquatic pollution or diagenetic overprint.

Environmental conditions are assessed with chemical analyses of water, sediment and organism soft tissue^18–21^. This type of monitoring is advantageous for short-term environmental condition evaluation, as it reveals its state when the water and soft tissue are sampled. Longer-term environmental conditions, such as an organism-derived response to environmental change and/or the impact of pollution on ecosystems, are not recorded with water, sediment and organism soft tissue analysis. This disadvantage can be overcome with the investigation of biomineralized hard tissue. As biologically secreted hard tissues record temporal trends of environment-derived chemical and physical change for their life-period, their structural materials are archives of environmental information, e.g. for pollution, diagenesis, environmental change. In particular, benthic foraminifera have been used as indictors of heavy mental pollution in the water. Munsel et al. (2010)^22^, Denoyelle et al. (2012)^23^, Ben-Eliahu et al. (2020)^24^ and Titelboim et al. (2021)^25^ used large benthic foraminifera for investigating water pollution and subjected *A. tepida*, *A. lobifera*, *A. lessonii* and *Sorites orbiculus* (Forsskål in Niebuhr, 1775) to waters with an increased heavy metal content. These studies showed that the above mentioned foraminifera species might be used as indicators of water pollution, as the shells incorporated the pollutants with a concentration that was comparable to the concentration of the pollutant in the water the specimens lived in. Nonetheless, the studies demonstrated as well that *A. tepida*, *A. lobifera*, *A. lessonii* and *S. orbiculus* are quite resilient to water polluted with Cd^2+^ and Pb^2+^; the foraminifera continued to generate chambers, even in strongly polluted water^20,22,23,26^. These results favour the notion that large benthic foraminifera can be used as biomarkers for seawater contamination with Ni, Cu, Mn, Cd^2+^ and Pb^2+^. They present, however, no abnormal tests as a result of heavy metal poisoning.

In this study, we experimentally demonstrate and discuss that the “crystal twin-related signal” of foraminiferal calcite reflects environmental perturbance and that it can be used as an indicator for a perturbed calcification environment, such as culture conditions with increased heavy metal concentrations. We characterize the calcite of *A. lessonii* and *A. lobifera* with Energy Dispersive Spectroscopy (EDS), Electron Backscatter Diffraction (EBSD) and FE-SEM imaging and describe for shells that were secreted in Cd^2+^- and Pb^2+^-unpolluted and polluted waters shell microstructure, texture, crystal co-orientation strength and twinned calcite generation. We find that: (i) The concentrations of cadmium (Cd^2+^) and lead (Pb^2+^) used in the culturing experiments do not significantly affect foraminiferal calcite microstructure, texture, or crystal co-orientation strength. Nonetheless, (ii) the used concentrations in Cd^2+^ and Pb^2+^ do influence the degree of calcite twin formation. For *A. lessonii*, relative to shell calcite secreted in uncontaminated conditions, the *addition of Cd*^*2+*^ causes the complete loss of twinned calcite formation, while, for *A. lobifera*, we see only a decrease in twinned calcite secretion. The *addition of Pb*^*2+*^ to the culturing environment generates a slight decrease in twinned calcite for *A. lessonii*, while for *A. lobifera*, the addition of Pb^2+^ to the culturing water initiates a dramatic decrease in the formation of twinned calcite.

Our study (i) shows that crystallographic characteristics of foraminiferal shell crystals might be used as pollution indicators and (ii) our results indicate that the metabolism of the foraminifera species may exert a different influence on the degree of twin formation in Cd^2+^- and/or Pb^2+^-contaminated water.

## Materials and methods

### Samples

This study investigates the following large benthic foraminifera species: *Amphistegina lessonii* (d’Orbigny, 1843) and *Amphistegina lobifera* (Larsen, 1976). *Amphistegina lobifera* was collected at 0.2–0.5 m water depths from an abrasion rock platform in the southeastern Mediterranean Sea. *Amphistegina lessonii* was sampled at 1–2 m water depths from rock pebbles in the Gulf of Aqaba-Eilat, Red Sea. Both locations are considered to be clean environments^24,26,27^.

### The culturing experiments

The investigated samples were obtained from culturing experiments performed and described in the study by Ben-Eliahu et al. (2020)^24^, aiming to test the applicability of larger benthic foraminifera for monitoring pollution in shallow marine environments. Of main interest was the determination of the ability of *A. lobifera* and *A. lessonii* to produce chambers in strongly poisoned environments with Cd^2+^ and Pb^2+^.

Culture vessels were kept in a temperature-controlled climate chamber, for a total of 24 days for *A. lessonii* and 31 days for *A. lobifera*. The temperature of the climate chamber was kept under the following conditions: 25 °C, 45 PAR (photosynthetically active radiation, µmol photons m^−2^ s^−1^) and 12-to- 12-hour light and dark cycles. Cd^2+^ and Pb^2+^ were then added to the culture water of the experimental group. 30 specimens were used per experiment for each culturing condition. The experiments had three replicates. After harvesting, samples were placed into tanks containing ambient sea water for recovery. After a few days, Cd^2+^ and Pb^2+^ were added to the culture water. Cd^2+^ and Pb^2+^enrichments in the culture water were increased, relative to the ecological criteria maximum concentration (CMC)^28,29^. In the case of Cd^2+^, for both species, the concentration was 4xCMC. For Pb^2+^, it was increased by 5xCMC for *A. lobifera* and 6xCMC for *A. lessonii.* Hence, Cd^2+^ addition was of 165–166 µg L^−1^ and Pb^2+^ addition was of 1001–1206 µg L − 1. The following number of chambers were grown in polluted conditions for each species: *A. lessonii* Cd^2*+*^ - 5 chambers; *A. lessonii* Pb^2+^ - 7 chambers; A. *lobifera* Cd^2+^ - 5 chambers; *A. lobifera* Pb^2+^- 5 chambers. Tracking of new chamber formation was done using a green calcein probe^24^.

### Sample preparation

**Fig. 1:**
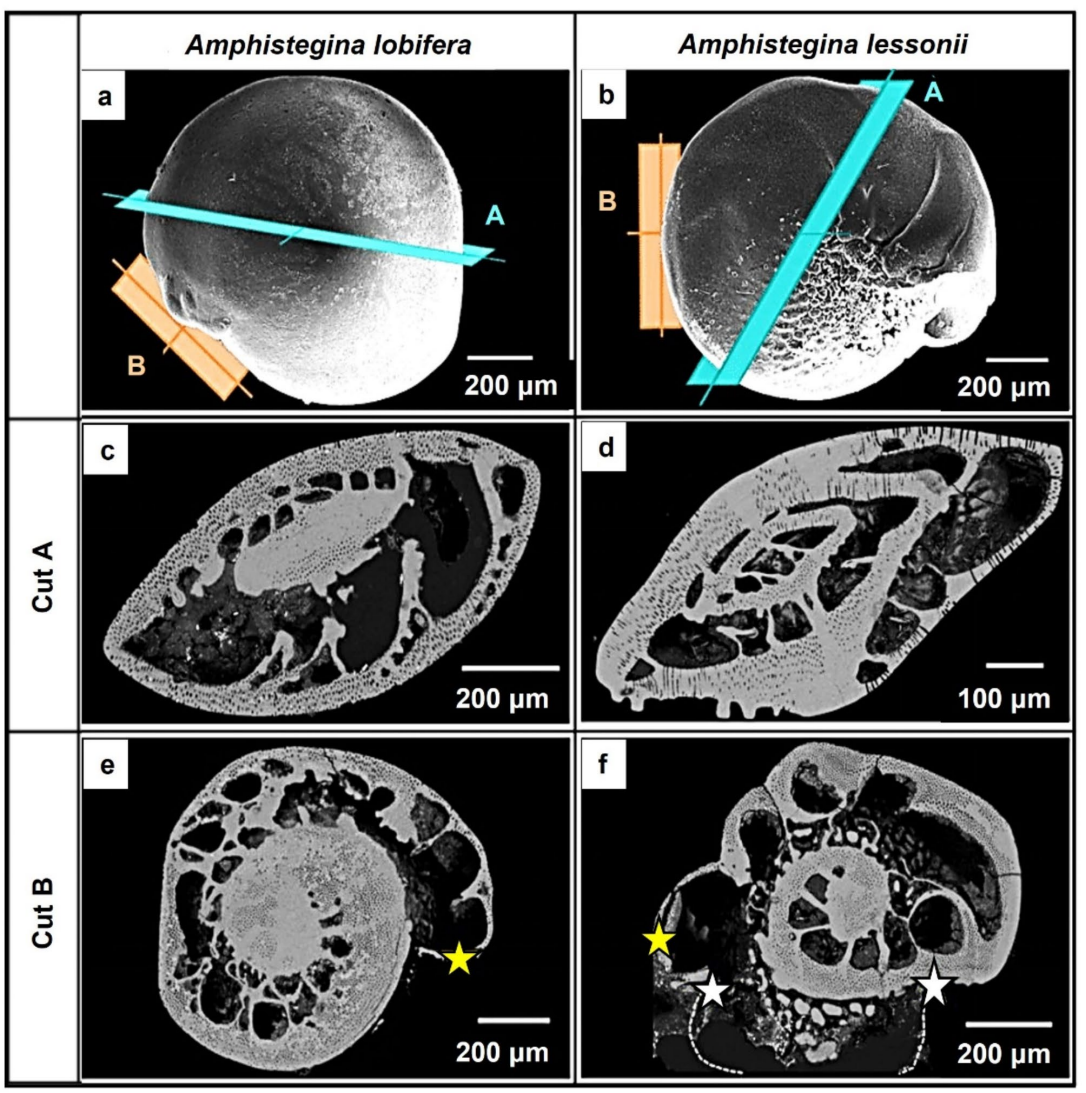
Shells of foraminifera species that were investigated in this study. The animals lived for their entire life in their natural marine environment. The structural characteristics of shells are used as reference for those organisms that were transferred into Cd^2+^ or Pb^2+^ contaminated water and secreted their last chambers in Cd^2+^ or Pb^2+^ environments. (A, B): SE micrographs of *A. lobifera* and *A. lessonii* shells. The reference shells were sectioned in two directions: cut A is a cross-section, cut B an equatorial section. (C) to (F): BSE images of the surfaces that were investigated with EBSD. In contrast to the first-formed chamber walls, the chamber walls that are formed as last (yellow star in (E), (F)) have thinner walls. The dashed white line and white stars in (F) indicate the position of the as last formed chamber wall; the latter became broken at microtome preparation. Modified after Fig. 1 in Yin et al. (2021)^6^.

**Fig. 2:**
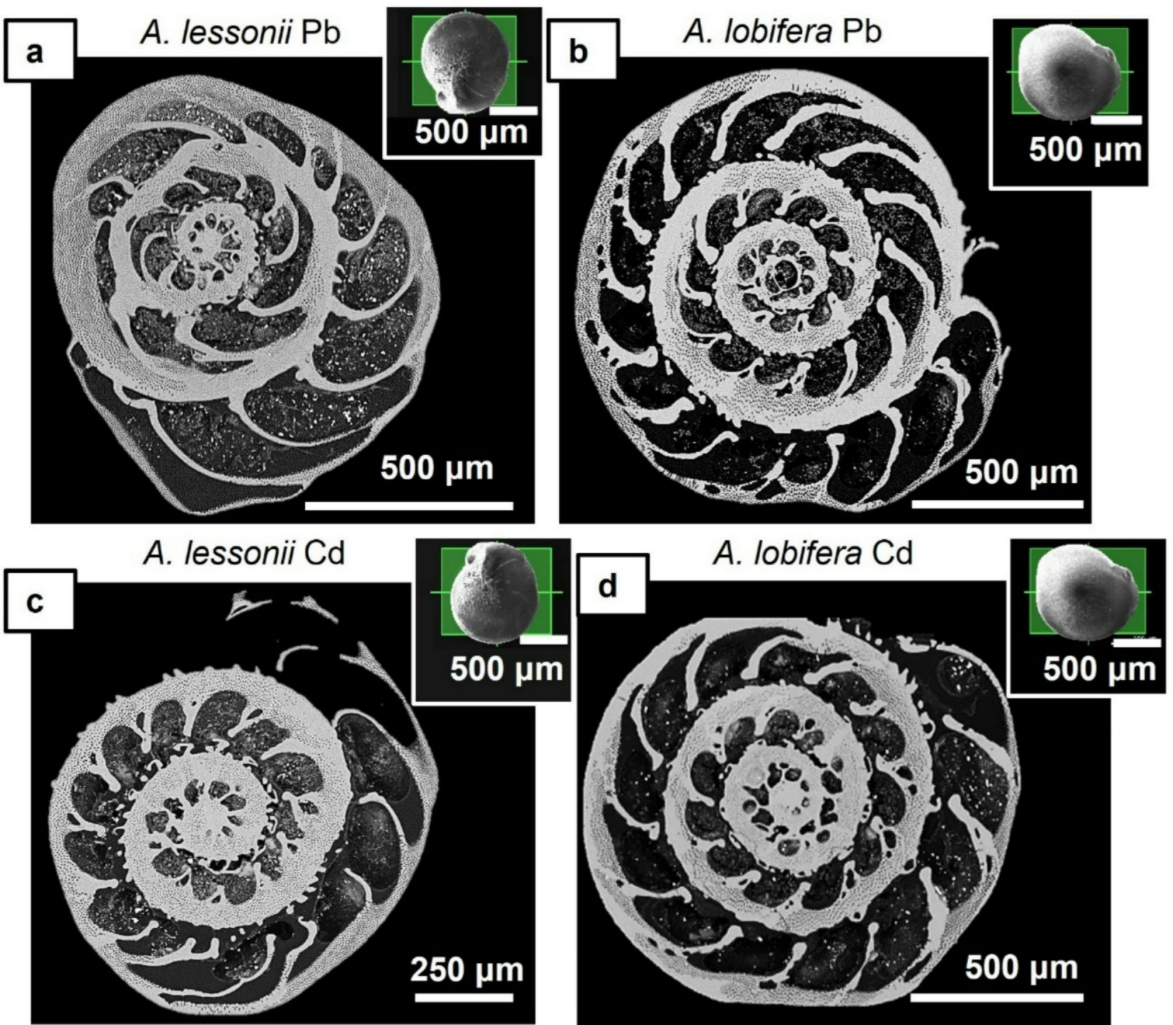
Shells of the foraminifera that lived first in their natural environment and, at a certain stage of growth, were transferred into tanks with Cd^2+^- or Pb^2+^-contaminated water. The last 5 to 7 chambers were secreted in Cd^2+^- or Pb^2+^-contaminated water. (A) to (D): BSE micrographs depicting an equatorial cut through the shell of *A. lessonii* ((A), (C)) and *A. lobifera* ((B), (D)). Inserts in (A) to (D) are SE images of the investigated shells.

In this study, we investigated reference shells of *A. lessonii* and *A. lobifera* (Fig. 1) cut in two directions, axially and equatorially^6^; species that were poisoned, cut equatorially (Fig. 2). For both species and for all types of samples (reference as well as poisoned), we investigated two specimens each. We performed in total 74 EBSD scans for this study. Considering *A. lessonii*, 18 scans were done on the *reference*, 11 scans on partly unpoisoned and partly *poisoned with Cd*^*2+*^ and 11 scans on partly unpoisoned and partly *poisoned with Pb*^*2+*^. On *A. lobifera* shells, we performed 15 scans on the *reference*, 9 scans on partly unpoisoned and partly *poisoned with* *Cd*^*2+*^ and 10 scans on partly unpoisoned and partly *poisoned with Pb*^*2+*^.

Two complete shells were investigated per species and type of experiment. One sample was attached with a minute drop of conductive glue onto an SEM-stub for imaging the overall appearance of the shell. The other sample was embedded into highly liquid EPON resin. After solidification of the resin, the shell and the EPON was cut and polished with an ultramicrotome for obtaining even sample surfaces (Figs. 1 and 2) such that, at a required 70° tilt for EBSD measurements, do not show any surface topography. For EBSD measurements the polished sample surfaces were coated with 4–6 nm of carbon while for SE and BSE imaging, they were coated with 4 to 8 nm of Pt/Pd.

### Methods

SE and BSE imaging, and EDS and EBSD measurements were carried out with a Hitachi SU5000 field emission SEM, equipped with an Oxford Instruments NordlyNano EBSD detector and an X-Max 80 × 80 EDS detector. EBSD scans were performed at 20 kV, with a step size of 150 to 250 nm. EBSD data was evaluated with the Oxford Instruments AZTEC and CHANNEL 5 HKL software and is presented as colour-coded crystal orientation maps, corresponding band contrast measurement maps and corresponding pole figures.

### Terminology

Subsequently, we define structural terms that we use in this study. For further information concerning the EBSD technique see Schwartz et al. (2000)^1^. For information related to twin formation, see other references^2,5,6,30,31^.

The *microstructure* of a crystallized material refers to the sizes, morphologies, co-orientation and misorientations, and modes of interlinkage of grains. It is shown with coloured EBSD maps. Similar colours reflect similar crystal orientations and different colours highlight differences in crystal orientation.

*Pole figures* are stereographic projections of crystallographic axes orientations measured for all pixels of an EBSD map or selected areas (subsets). The viewing direction of the pole figures is the same as the viewing direction of the corresponding EBSD maps. All pole figures shown in this study display the lower hemisphere. This ensures that the pole figures are displayed in the same spatial orientation as the corresponding EBSD map. With pole figures we either show individual orientation data points or the density distributions of the orientation data.

The *texture* or *crystallographic preferred orientation* of a crystallized substance relates to the distribution of all crystal orientations within a material and is shown with pole figures. A single-crystal-like texture is present when clear-cut maxima are observed in the pole figures of all crystallographic axes. Accordingly, for calcite, we need to observe one cluster for the c-axes and three clusters for the a*-axes in the pole figure. An axial/cylindrical texture is developed in the relevant material when the c-axes show a cluster in one particular direction and the orientation of the a*-axes scatter on a great circle perpendicular to the c-axis cluster.

*Crystal co-orientation statistics* are derived from Kikuchi patterns measured at each pixel of an EBSD map. The degree of calcite co-orientation within individual crystals is obtained from measurements of the orientational density distribution, the multiple of uniform (random) distribution (MUD) value.

The *MUD* is calculated by the CHANNEL 5 EBSD software and is an indication of the strength of crystal co-orientation. A high MUD indicates high crystal co-orientation and low MUD values indicate low to random crystallite and/or mineral unit co-orientation. The parameters for data contouring in the pole figures were fixed to a half width of 5 and a cluster size of 3. For these parameters, a MUD value of 700 and higher indicates single crystallinity, while a MUD value of 1 indicates poly-crystallinity.

The *EBSD band contrast measurement map* depicts the signal strength of the Kikuchi pattern at each measurement point in the EBSD scan. It is displayed as a grey-scale component in the map. White to light grey colours indicate a high intensity of the Kikuchi signal, corresponding to strong mineralisation, while dark grey and black colours point to a weak or absent Kikuchi signal, e.g. when organic matter, resin or voids are scanned.

*Twinned crystals* are entities, where adjacent crystals of the same phase are intergrown in a regularly recurring orientational relationship, e.g. reflection, rotation or inversion. These induce different crystal orientation states and generate the *twin domains* of a *twinned crystal*. The orientational relationship between twin domains is defined by the *twin laws*. The twin domains of a twin crystal are separated from each other by twin planes. In contrast to inorganic materials, in biological materials twins do not show perfectly planar and parallel twin planes.

For biologically secreted carbonate structural materials, growth or deformation twins are reported. Growth twins are intrinsic to the material in question and are generated at hard tissue formation (e.g^2,3,6^). Deformation twins are the result of external, natural or artificial, impact on the material in question^32,33^. Growth twins belong to the family of crystal growth defects which are the result of an accidental departure from the condition of minimal Gibbs free energy of an untwinned crystal individual^34,35^. As at nucleation and growth often supersaturation conditions and, thus, high nucleation rates prevail, a group of atoms can take a subminimum energy position^36^. This may result in the generation of a second crystal individual on the surface of a first and this (second) crystal individual is in twin relation to the original crystal individual. Before the atoms/molecules of the second crystal individual move to a minimum energy position that corresponds to that of the untwinned crystal, the second crystal starts to grow in an alternative position and with a different orientation. In this case, the original crystal and the second crystal individual become the twin domains of a twin crystal^34,35^.

We prove the presence of twinned calcite with the specific *misorientation boundary* for the twin law. For calcite, the *misorientation at the twin boundary* is around *60°*. We show the characteristic peak for this in the *misorientation angle distribution diagram*. From this diagram, the value for the frequency at the 60*°* peak was extracted to obtain values for measurements in areas grown under unpoisoned conditions and measurements in areas grown under poisoned conditions. These frequency values were then separated into these two groups and an average was taken, with the corresponding standard deviation. This data was also plotted using *box and whisker plots* to illustrate further the frequency spread under the different growth conditions.

## Results

**Fig. 3:**
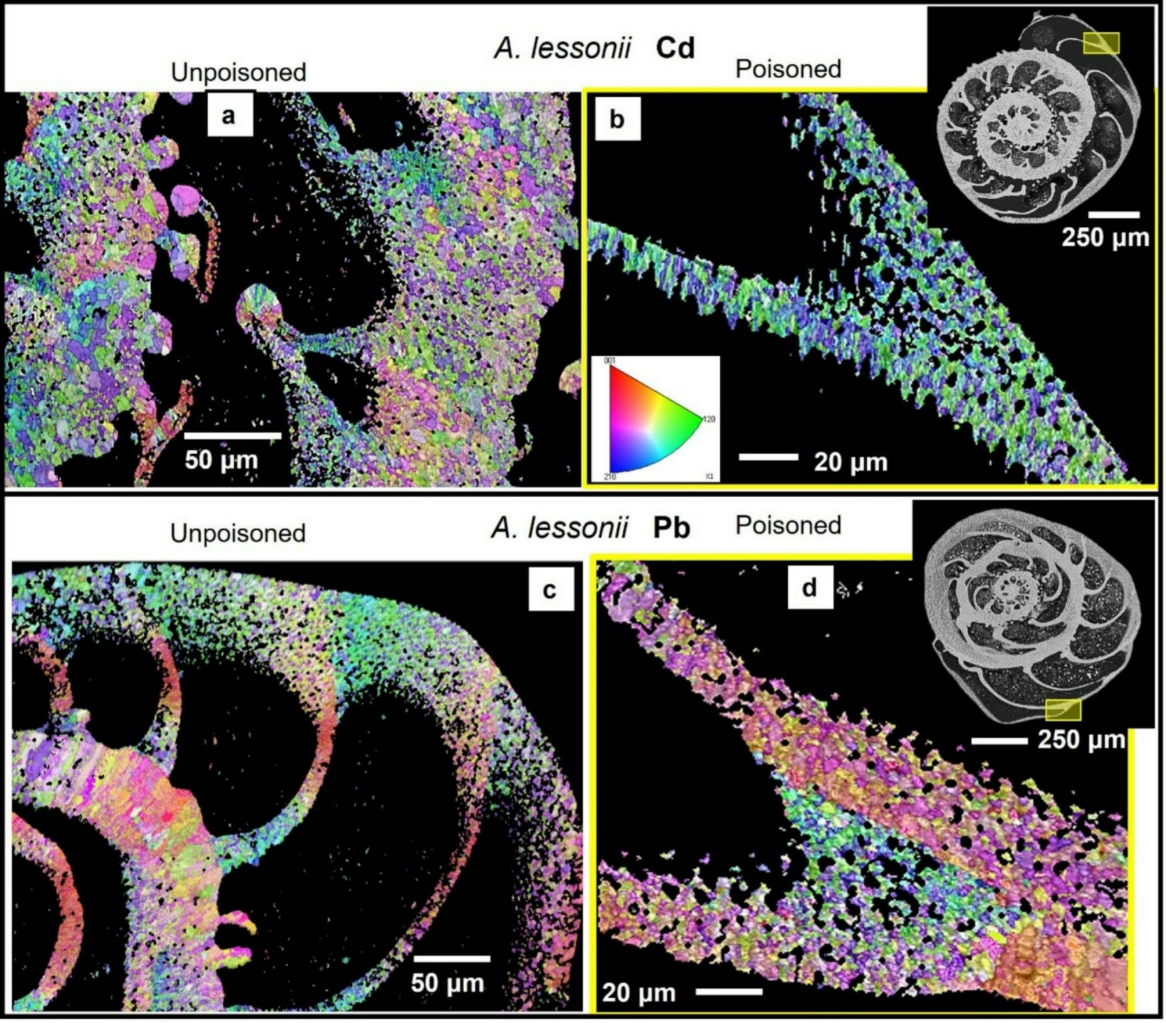
Shell microstructure of *A. lessonii* specimens that lived first in natural environments ((A), (C)) and, for the secretion of the last few chambers, lived in water contaminated with either Cd^2+^ or Pb^2+^ ((B), (D)). (A) to (D): EBSD scans showing crystal orientation. Yellow rectangles in inserts in (B), (D) indicate the position of EBSD scans shown in (B) and (D). In those shells where calcite growth takes place entirely in natural environments, the morphology of crystals within the at last formed chambers is euhedral and crystal sizes are in the very few micrometer range (this study and Fig. 11 in Yin et al. (2021)^6^). This is also the case at last secreted chamber walls of species that were secreted in polluted environments ((B), (D)). There is no marked difference in microstructure, crystal size or crystal morphology between shell sections secreted under natural and under poisoned conditions.

**Fig. 4:**
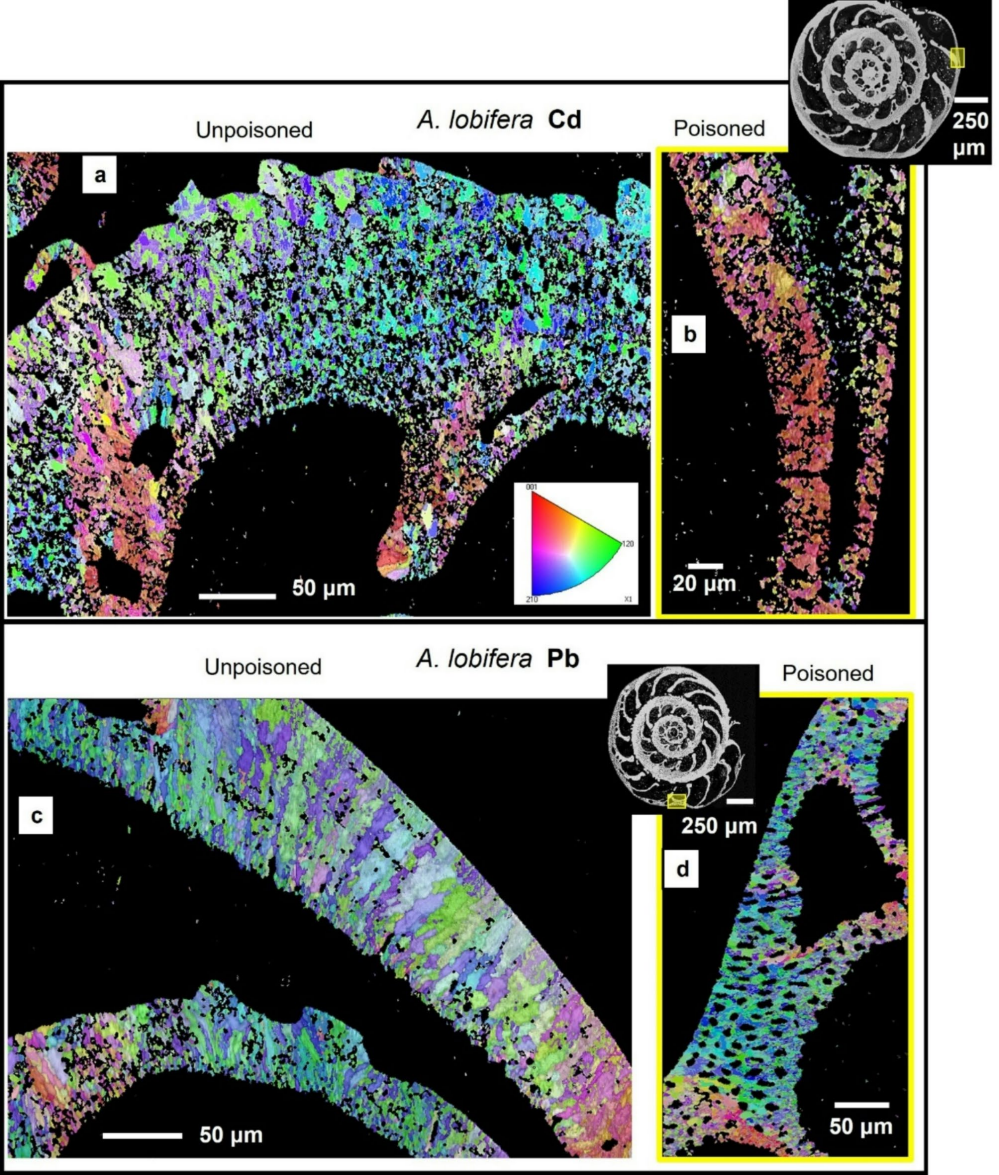
Shell microstructure of *A. lobifera* specimens that lived first in natural environments ((A), (C)) and, for the secretion of the last few chambers, were transferred into water contaminated with either Cd^2+^ or Pb^2+^ ((B), (D)). (A) to (D): EBSD scans showing crystal orientation. Yellow rectangles in inserts in (B), (D) indicate the positions of EBSD scans shown in (B), (D). (B) shows the shell wall of the last chamber, (D) shows the shell wall of the last but fifth chamber. There is no marked difference in microstructure between shell sections secreted under natural and under Cd^2+^- or Pb^2+^-poisoned conditions.

Figure 1 shows the investigated *A. lobifera* and *A. lessonii* shells and the mode of sectioning through the shells. These species lived for their entire life in Cd^2+^- and Pb^2+^-uncontaminated environments. Figure 2 depicts the shells of species that lived first in their natural environment and, at some point, were transferred to tanks, survived, and carried on secreting chambers in Cd^2+^ and Pb^2+^ contaminated water. When the overall shell structure, chamber wall thickness, regularity of chamber wall thickness for the last chambers that were secreted in the different environments is compared, we do not find any noticeable structural difference. This is also the case for shell microstructure, shown in Figs. 3 and 4, and determined from EBSD measurements. Figures 3 and 4 depict shell crystal organization for *A. lessonii* and *A. lobifera*, secreted under unpoisoned (Figs. 3A and C and 4A and C) and poisoned (Figs. 3B and D and 4B and D) environments. There is no difference observable in crystal size and/or crystal morphology. Hence, based on these characteristics, we do not find any structural difference of calcite crystals secreted in unpoisoned and poisoned waters. For band contrast measurements of these sections, please see Fig. S1 and Fig. S2 for *A. lessonii* and *A. lobifera*, respectively. The incorporation of Pb^2+^ and Cd^2+^ into the shells was checked with EDS measurements, with both map measurements and energy spectra. Figures S3 and S4 show this for Pb^2+^-poisoning.

**Fig. 5:**
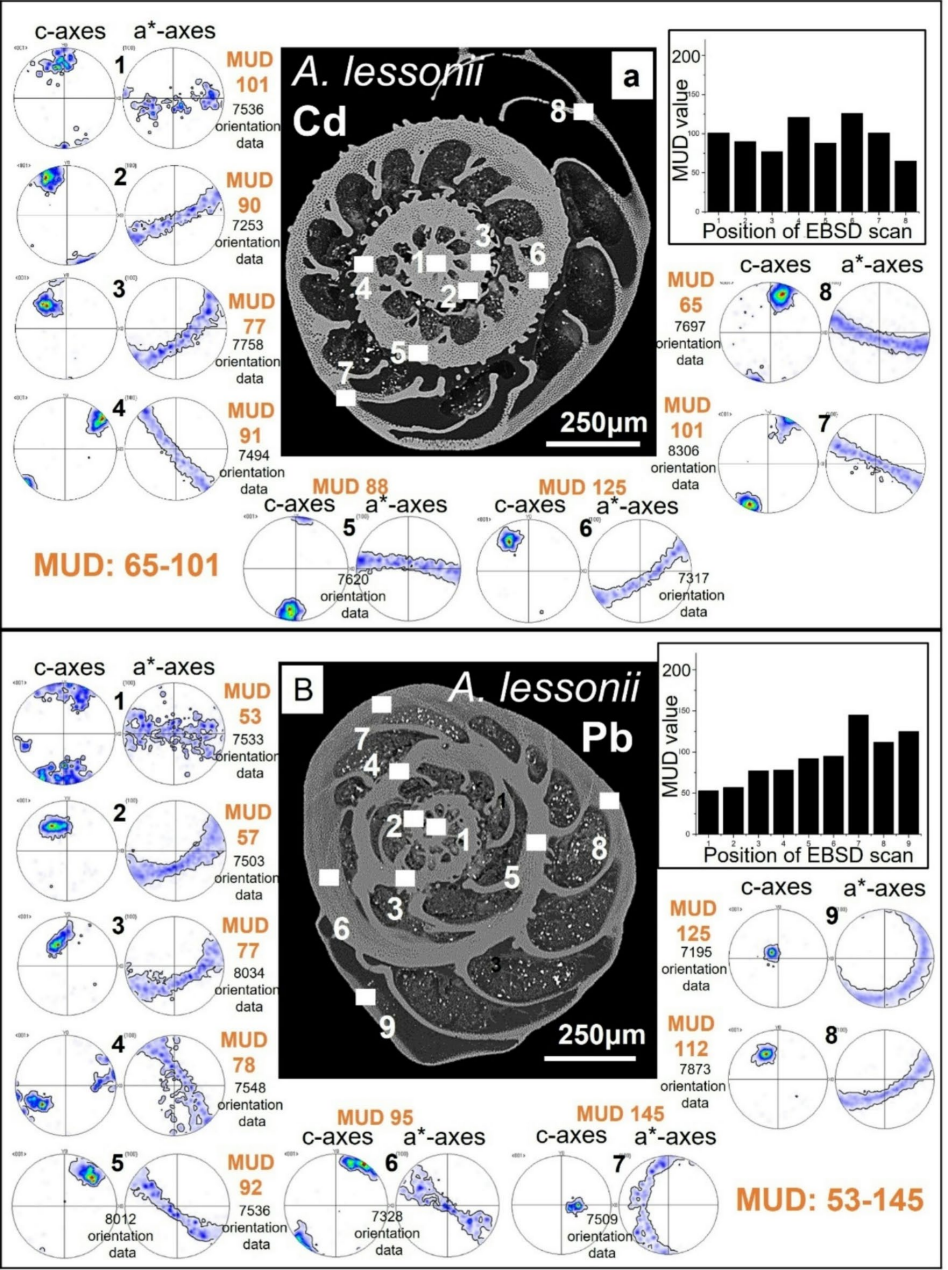
The texture and crystal co-orientation strength of *A. lessonii* shell calcite. (A): Water contaminated with Cd^2+^; (B): water contaminated with Pb^2+^. In (A) measurements 7 and 8 are taken on poisoned shell sections, in (B) measurements 8 and 9 are taken on poisoned shell sections. As the pole figures show, we find for all measurements an axial texture for shell calcite. For all EBSD scans calcite c-axis is perpendicular to outer shell surface and rotates with the curvature of the shell. There is no difference in type of texture or calcite c-axis orientation between chambers secreted in a natural environment and chambers secreted in polluted water. Crystal co-orientation strength is increased, see MUD values in (A), (B). There is a slight increase in crystal co-orientation strength between for the species shown in (B).

**Fig. 6:**
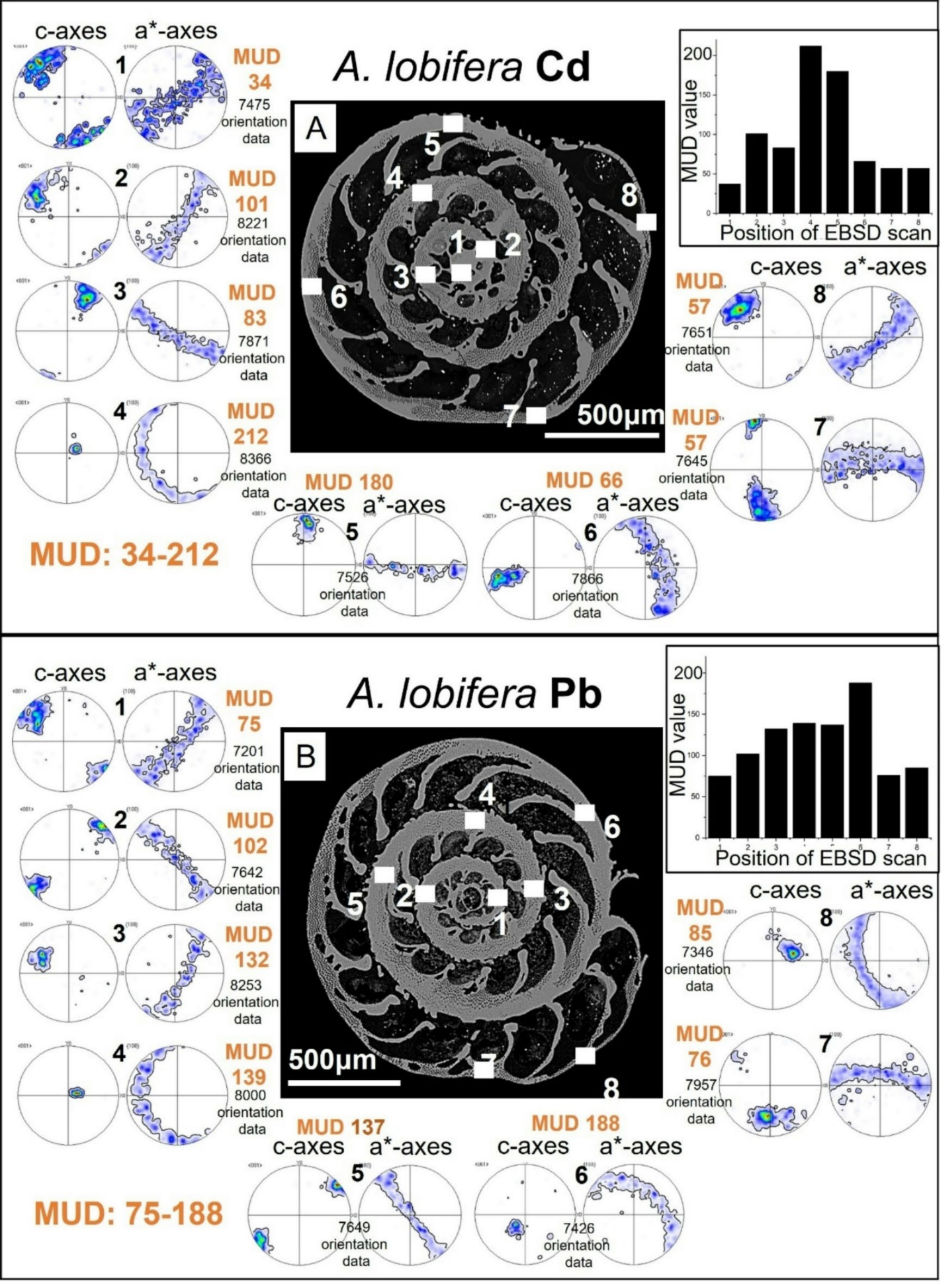
The texture and crystal co-orientation strength of *A. lobifera* shell calcite. (A): Sample contaminated with Cd^2+^; (B): sample contaminated with Pb^2+^. Measurements 7 and 8 are taken on poisoned shell sections. As the pole figures show, we find for all measurements an axial texture for shell calcite. For all EBSD scans calcite c-axis is perpendicular to outer shell surface and rotates with the curvature of the shell. There is no difference in type of texture or calcite c-axis orientation between chambers secreted in a natural environment and chambers secreted in polluted water. Crystal co-orientation strength is quite increased, see MUD values in (A), (B). We find for *A. lobifera* a higher crystal co-orientation strength, relative to that for *A. lessonii*; compare MUD values of Figs. 5 and 6.

Figures 5 and 6 depict for *A. lessonii* (Fig. 5) and *A. lobifera* (Fig. 6) crystal co-orientation strength, expressed with MUD values, and calcite texture, expressed with pole figures, of up to nine EBSD scans, taken on different locations of the shell cross-sections. Measurements 1 to 6 were taken on shell portions where the organisms lived in Cd^2+^- and Pb^2+^-uncontaminated waters, while measurements 7 and 8 were recorded on shell sections that were secreted in Cd^2+^- and Pb^2+^- contaminated waters. We find for both investigated species: (i) for all EBSD scans an axial texture, an axial preferred orientation of crystals, and (ii) no distinct difference in crystal co-orientation strength (MUD value) for measurements on shell portions formed in uncontaminated and contaminated environments. The MUD values of EBSD measurements for both species show a large scatter and do not show a clear-cut difference for measurements conducted on shell sections secreted in unpoisoned and poisoned environments. We also do not see any reasonable clear-cut trend in crystal orientation strength for the different EBSD scans performed on the shells of the two investigated foraminifera species. In essence, shell material structure-related parameters such as microstructure, texture and crystal co-orientation strength allow no distinction between shell portions mineralized in uncontaminated and contaminated environments (Figs. 1, 2, 3, 4, 5 and 6).

**Fig. 7:**
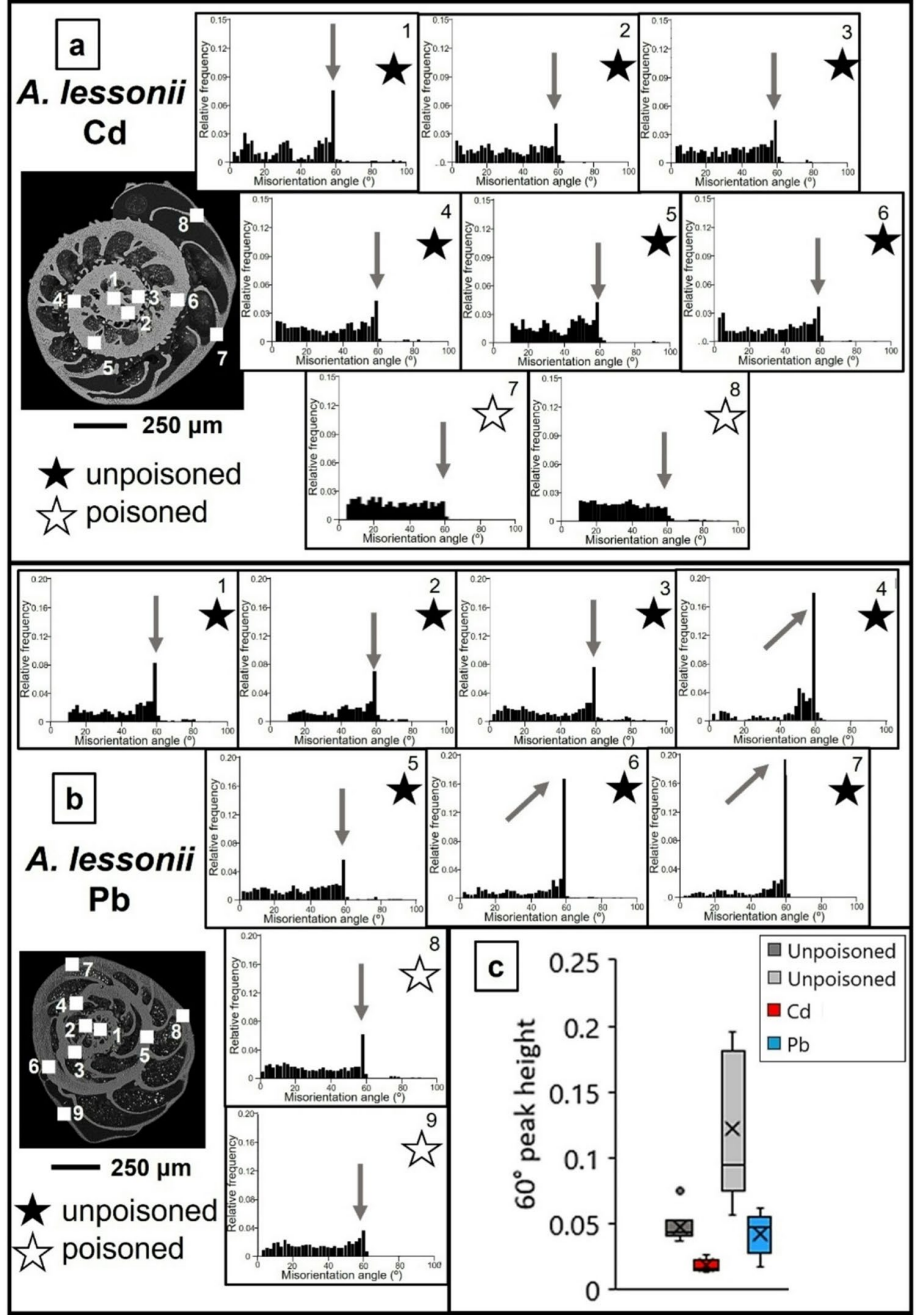
The presence of the 60° misorientation peak in relative frequency versus misorientation angle diagrams for *A. lessonii* shells. The diagrams are deduced from EBSD scans; the positions of these are shown in inserts in (A) and (B). EBSD measurements are taken on different sections of the shell wall. The last two scans are taken on chamber walls that were secreted in Cd^2+^- and/or Pb^2+^-contaminated water (white star). Diagrams marked with a black star represent shell portions that formed in uncontaminated water. We observe in (A) and (B) the strong reduction of the 60° misorientation peak for shell portions that grew in contaminated environments, however, also a scatter in the degree of twin formation for those shell parts that formed in uncontaminated waters. We find a difference in 60° misorientation peak height for the species shown in (A) and in (B). For those shell sections that formed in unpolluted waters, twinned calcite is present. Those shell sections that formed in waters polluted with Cd^2+^ or Pb^2+^ contain significantly less or possibly no twinned calcite. (C) Box-whisker plot statistically showing the comparison between the 60° peak in shell areas grown in unpoisoned and poisoned areas. Poisoning appears to decrease the height of the peak for both elements.

**Fig. 8:**
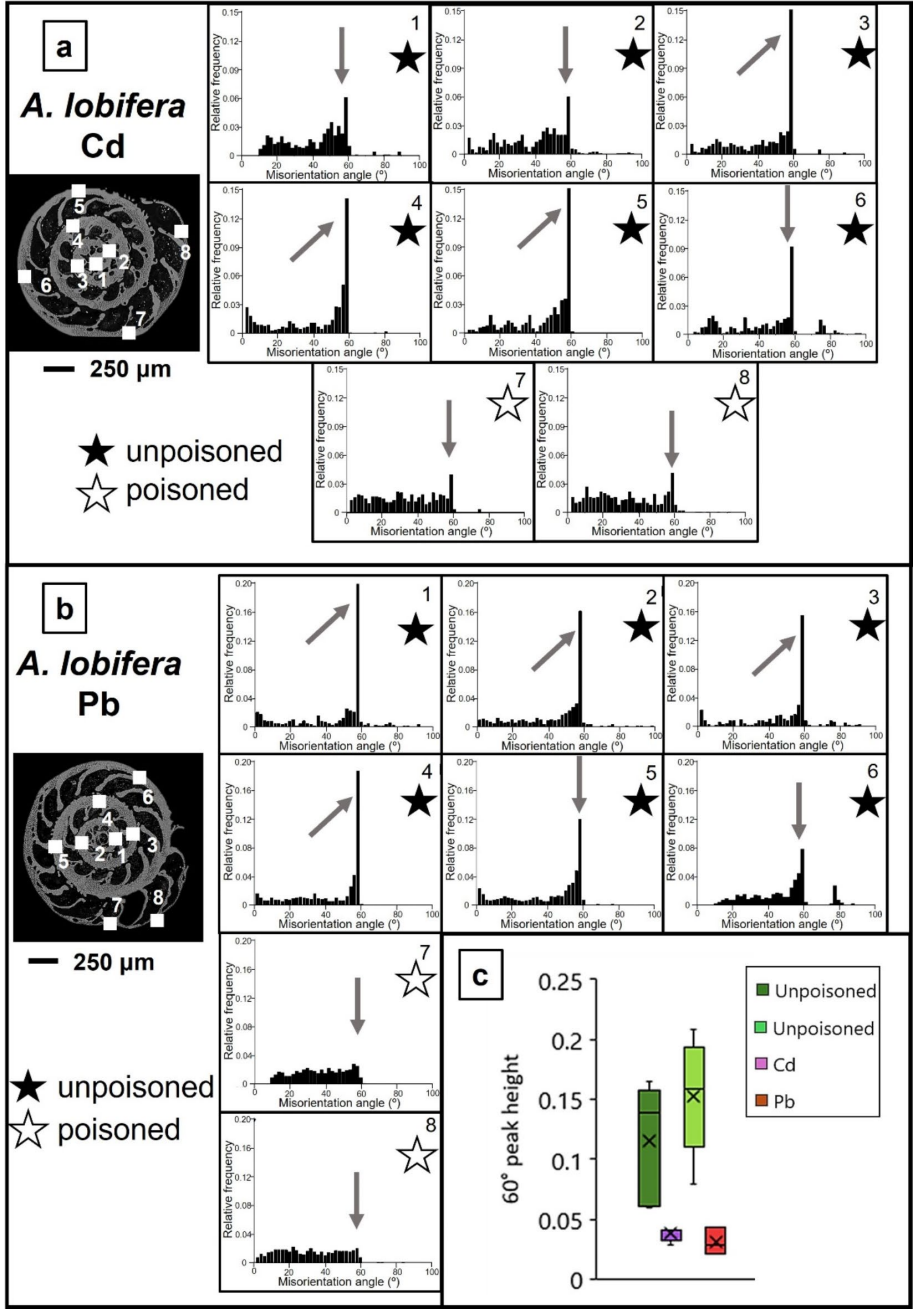
The presence of the 60° misorientation peak in relative frequency versus misorientation angle diagrams for *A. lobifera* shells. The diagrams are deduced from EBSD scans; the position of these are shown in inserts in (A) and (B). EBSD measurements are taken on different sections of the shell wall. The last two scans are taken on chamber walls that were secreted in Cd^2+^ or Pb^2+^-contaminated water (white star). Diagrams marked with a black star represent shell portions that formed in uncontaminated water. The 60° misorientation peak indicates the presence of twinned calcite in the shell. With the exception of the last two measurements that are taken on shell portions that formed in polluted water, we find a peak at 60° misorientation. The latter is significantly reduced for shell sections that grew in water contaminated with Cd^2+^ or Pb^2+^. The comparison between Figs. 7 and 8 shows a higher 60° misorientation peak for *A. lobifera*. (C) Box-whisker plot statistically showing the comparison between the 60° peak in shell areas grown in unpoisoned and poisoned areas. Poisoning appears to decrease the height of the peak for both elements.

Figures 7 and 8 show relative frequency versus misorientation angle diagrams for the investigated species. These are deduced from EBSD scans and are shown for all the scans taken on the surface of the cut through the shell. We find in most diagrams a marked peak at 60° misorientation (pointed to by the arrow in the relative frequency – misorientation angle diagrams). For both species, irrespective whether Cd^2+^ or Pb^2+^ was used for poisoning, we find a marked decrease in the 60° peak in shell sections that were secreted under poisoned conditions, relative to the 60° peak in those shell parts that formed in uncontaminated environments. For *A. lessonii* poisoned with Cd^2+^, the unpoisoned area averages a 60° misorientation peak height of 0.05(1) while those grown in poisoned environments average 0.018(5). In the case of Pb^2+^, these values are of 0.12(5) and 0.04(1), respectively. For *A. lobifera*, the unpoisoned area averages a 60° misorientation peak height of 0.12(5) while those grown in poisoned environments average 0.038(5). In the case of Pb^2+^, these values are of 0.15(4) and 0.031(9), respectively. This is further illustrated in the box and whisker plots seen in Figs. 7c and 8c. The 60° peak in the relative frequency-misorientation diagram indicates the formation of twinning in the shells. The more marked the 60° peak is in the diagram, the more extensive the presence of twinned calcite in the shell. A lack of the 60° peak in the relative frequency-misorientation angle diagram indicates the lack of twinned calcite in the shell.

**Fig. 9:**
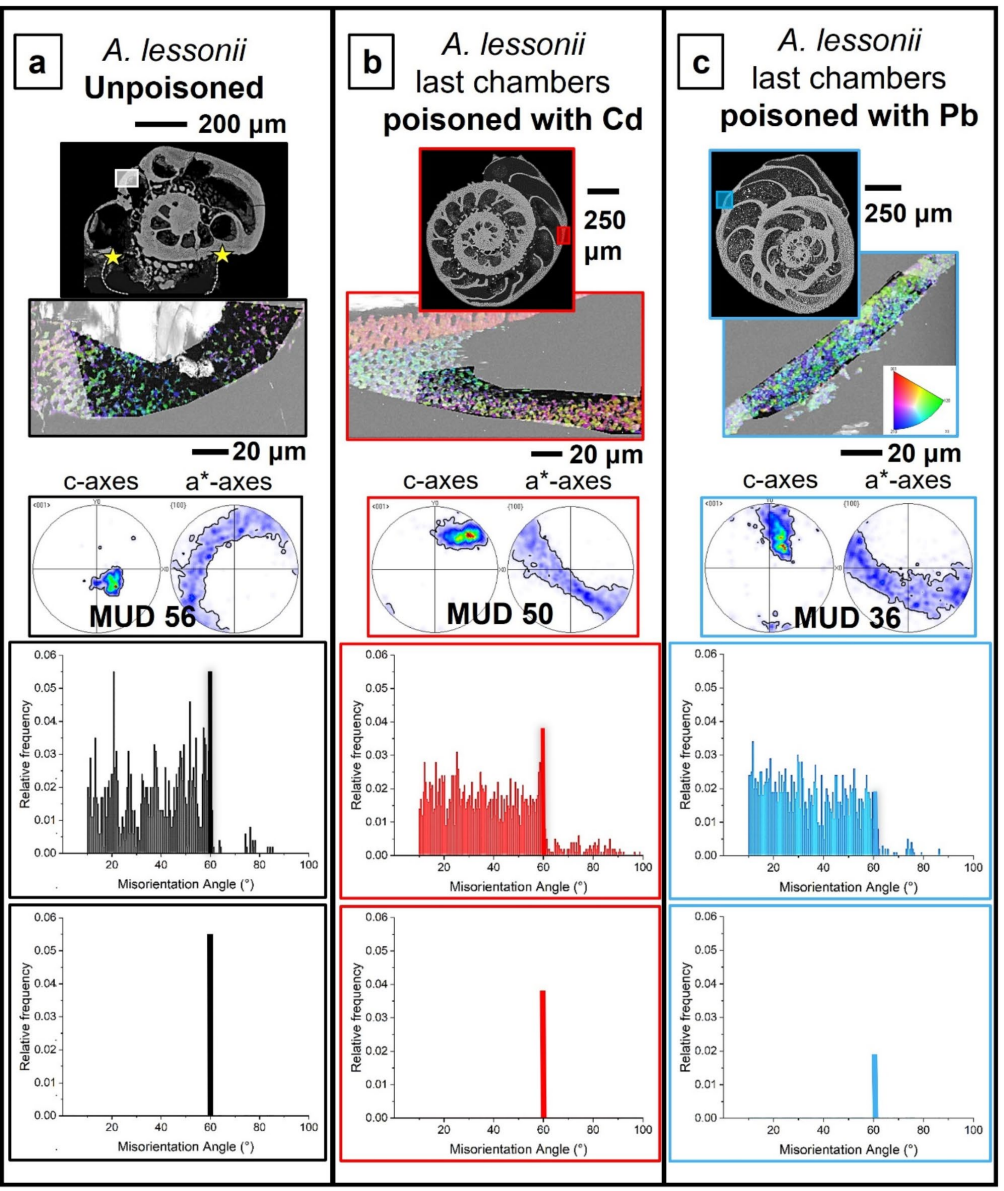
Shell texture, crystal co-orientation strength and twinning characteristics for *A. lessonii* chamber walls secreted in uncontaminated (A), and in Cd^2+^- (B) and Pb^2+^- (C) contaminated waters. We show the EBSD scan, the pole figures and MUD value for the latter, the misorientations that we find in the shown scan and its 60° misorientation peak. The position of EBSD scans is indicated with white rectangles on the cuts through the shell. When compared to the shell that formed in Cd^2+^- and Pb^2+^-uncontaminated environments, we find a slight decrease in crystal co-orientation strength, a marked decrease in the 60° peak in the relative frequency-misorientation angle diagram and little to no change in degree and range of the other misorientations. We find also that contamination of water with Pb^2+^ has a stronger effect on inhibiting twin formation, relative to the effect of Cd^2+^. The 60° misorientation peak of the chamber that was secreted in Pb^2+^-polluted water is lower than for that formed in Cd^2+^-polluted water.

**Fig. 10:**
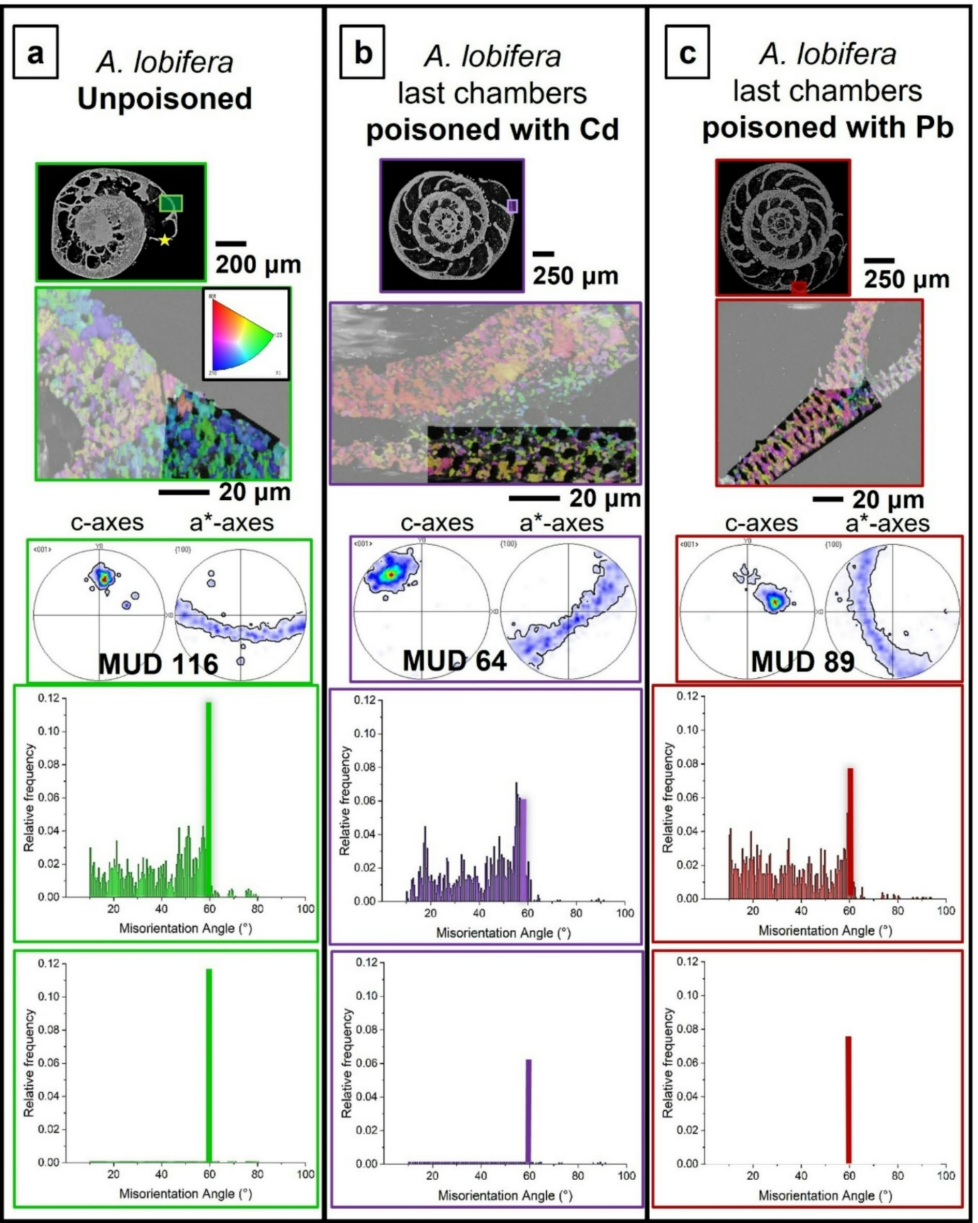
Shell texture, crystal co-orientation strength and twinning characteristics for *A. lobifera* chamber walls secreted in uncontaminated (A), and in Cd^2+^- (B) and Pb^2+^- (C) contaminated waters. We show the EBSD scan, the pole figures and MUD value for the latter, the misorientations that we find in the shown scan and its 60° misorientation peak. The position of EBSD scans is indicated with white rectangles on the cuts through the shell. When compared to the shell that formed in Cd^2+^- and Pb^2+^-uncontaminated environments, we find a decrease in crystal co-orientation strength, a decrease in the 60° peak in the relative frequency-misorientation angle diagram and little to no change in degree and range of the other misorientations. In contrast to the effect of Cd^2+^ and Pb^2+^ contamination on *A. lessonii* shells, as for A. *lobifera*, the influence of Cd^2+^ and Pb^2+^ on twin formation is not as substantial. Furthermore, we do not see such a marked difference in the height of the 60° misorientation peak for Cd^2+^ and Pb^2+^.

The effect of chamber growth in poisoned environments is further illustrated in Figs. 9 and 10. A reference sample is presented and compared with the specimens grown under poisoned conditions for both species, in order to also compare chambers of similar ages. The 60° peak was extracted and presented for a clearer comparison. There is a clear decrease in the peak upon poisoning. There is also a decrease in MUD values, more marked for *A. lobifera.*

## Discussion

### Biomineralization

Biomineralization is the formation of mineralized tissue resulting from biological activity. It is a constructive process where organisms interact with the inorganic realm of their environment for the generation of mineralized structural materials. These are fabricated by organisms for a wide range of tasks, of which protection is the most prominent one.

In marine environments, carbonate biominerals incorporate calcium, magnesium, carbon and oxygen as well as ions which substitute in the biocrystals for either Ca^2+^ or CO_3_^2−^. The elemental and/or isotopic composition of the crystals that comprise the substitutes, reflect, at least to some degree, the environment the organisms live in, e.g. the chemistry, temperature and salinity of the seawater (e.g^37^). Hence, biomineralized hard tissues are biogenic archives of environmental information, of, among others, ocean water temperature, salinity and pH variations as well as water pollution (e.g^25,26,37–40^). In particular, water pollution can be monitored with Ca-carbonate structural materials. This has been shown by the studies of Munsel et al. (2010)^22^, Denoyelle et al. (2012)^23^, Ben-Eliahu et al. (2020)^24^and Titelboim et al. (2021)^25^. *Ammonia tepida*, *Amphistegina lobifera*, *Amphistegina lessonii* and *Sorites orbiculus* (Forsskål in Niebuhr, 1775) were subjected to waters with an increased heavy metal content. The studies showed that these foraminifera species could be used as indicators of water pollution, as the shells incorporated the pollutants with a concentration that was comparable to the concentration of the pollutant in the water the specimens lived in. The above mentioned studies demonstrated, however, as well that *A. tepida*, *A. lobifera*, *A. lessonii* and *S. orbiculus* are quite resilient to water polluted with Cd^2+^ and Pb^2+^, as the foraminifera continued to generate chambers, even in strongly polluted water^20,24,26,27^. Hence, these results favour the notion that the calcite of large benthic foraminifera shells can be used as biological markers for seawater contamination with Ni, Cu, Mn, Cd^2+^ and Pb^2+^.

Nonetheless, ion substitution does not initiate substantial changes of atomic positions in the crystal structure of calcite, our study demonstrates that one particular structural characteristic of calcite crystals is strongly affected: the formation of crystal twinning. We take advantage of the fact that rotaliid foraminifera secrete twinned calcite for shell formation and show that the twinned nature of the calcite is affected by the addition of Pb^2+^ and Cd^2+^ to ambient water. Our study proposes the twin characteristic of the foraminiferal calcite as a structural biomarker for monitoring water contamination with Pb^2+^ and Cd^2+^ and, possibly, other heavy metal elements.

### What is a twinned crystal?

A crystal twin is a 2-dimensional defect that separates two parts of a single crystal in a symmetric way^41^. Generation of twinned crystals takes place at (i) nucleation and crystal growth, (ii) at shear deformation and (iii) at transformation during a phase transition (e.g. Hahn and Klapper (2006)^30^). Accordingly, based on their genesis, crystal twins are categorized as growth, deformation or transformation twins^30,35,41^. For biologically secreted Ca-carbonate hard tissues growth and deformation twins are reported. Growth twins are intrinsic to the material in question, as they are generated at hard tissue formation (e.g^2,3,6^)., while deformation twins are the result of external impact on the material in question^32,33^.

In a twinned crystal, two or more domains of the same species and similar chemical composition intergrow. The intergrowth of twin domains is related to each other in a symmetrical fashion and is defined by crystallographic operation determinants, the twin laws^13,42–45^. The twin domains of a twin crystal share some crystal lattice points and, along these, are tightly bonded to each other. Accordingly, the shared lattice points give the junction between the twin domains a much greater strength, relative to a junction that is given between randomly oriented grains. This interface between twin domains may be planar and coincide with the mirror-plane of the twin law. This is the case if the twin domains are related by a shear deformation (for details see^30^), however, in general, and in the present case of the 60° < 001 > twins, there is no such constraint on the interface.

The interface between adjacent twin domains is a unique structure and is diagnostic for the crystal twin in question^46–48^. As twin interfaces are the result of a structural relationship between adjacent twin domains, the orientation of twin domains at the twin interface is inherent for a twin crystal^48^. The structural relationship between twin domains is a crystallographically-defined symmetry operation. It is either a reflection, a rotation or an inversion operation^30^ and is given with the twin law in question. For inorganic calcite five twin laws are known, named after the Miller indices of the twin planes ((001), (018), (104), (012), (108)). For all calcite twin laws the twin plane is a mirror plane and adjacent twin domains are misoriented to each other by 60° for the (001) twin, by 78.1° for the (018) twin, by 103.9° for the (104) twin, by 78.8° for the (012) twin and by 78.1° for the (108) twin^43,46,49,50^and Table 2 in Schmahl et al. (2024)^5^. Recently, Schmahl et al. (2024)^5^characterized, for foraminiferal shell calcite, a new, non-classical, but systematically reoccurring, twin-related, misorientation. For inorganic aragonite, so far, one twin law is reported. The twin plane in an aragonitic twinned crystal is a glide plane parallel to (110) and adjacent twin domains are misoriented to each other by 63.8°^13,44,50^.

**Fig. 11:**
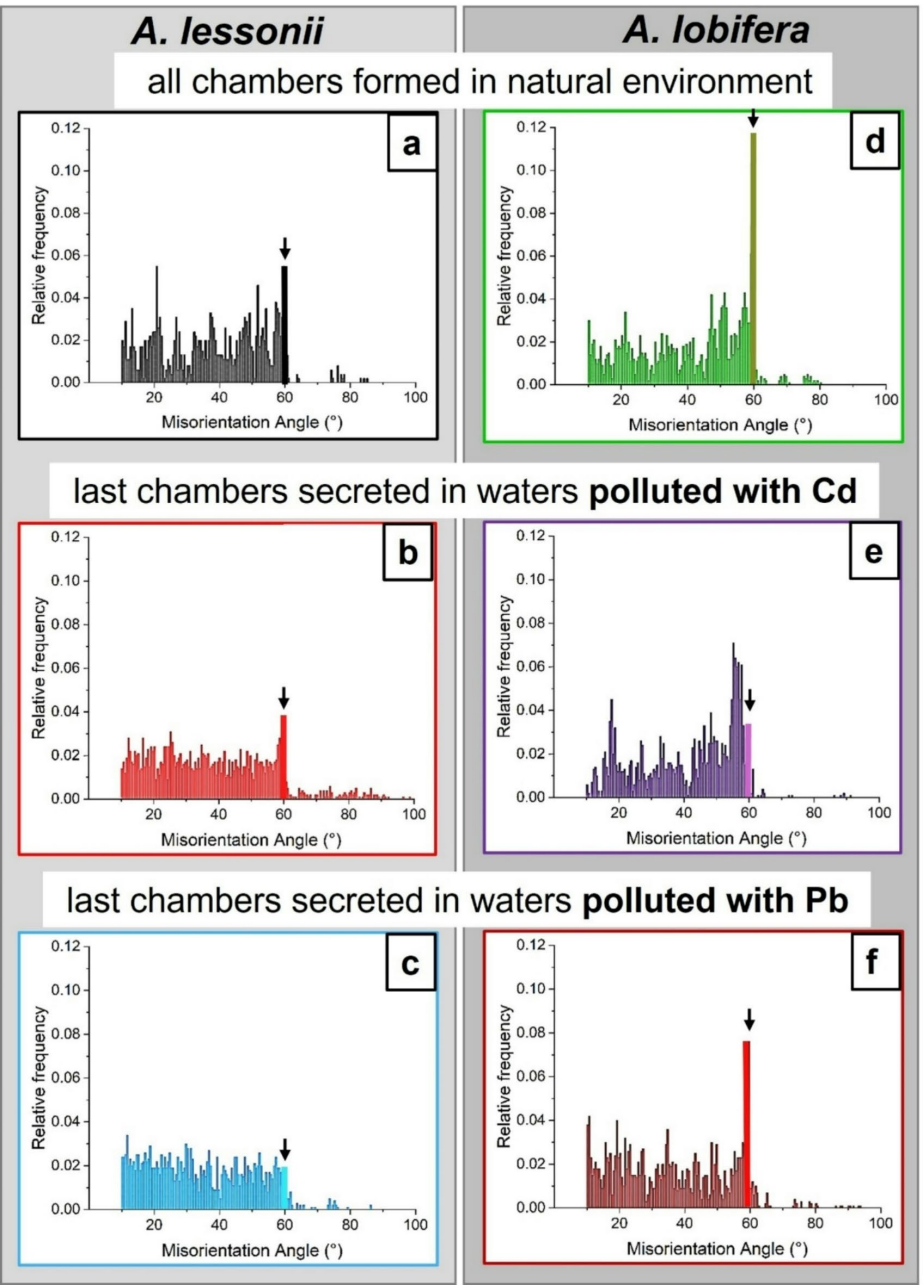
The range and strength of misorientations between chamber wall crystals for *A. lessonii* and *A. lobifera*. The shells were secreted first in Cd^2+^- and Pb^2+^-uncontaminated water ((A), (D)). The last chambers were formed in Pb^2+^- and Cd^2+^-contaminated water (Fig. 11B, C, E, F). The only difference that we find is in the 60° misorientation. For the other misorientations, there is no marked difference in range and strength between them. However, the difference in 60° misorientation is striking. For a closer look at the 60° misorientation peak, see Fig. 12.

**Fig. 12:**
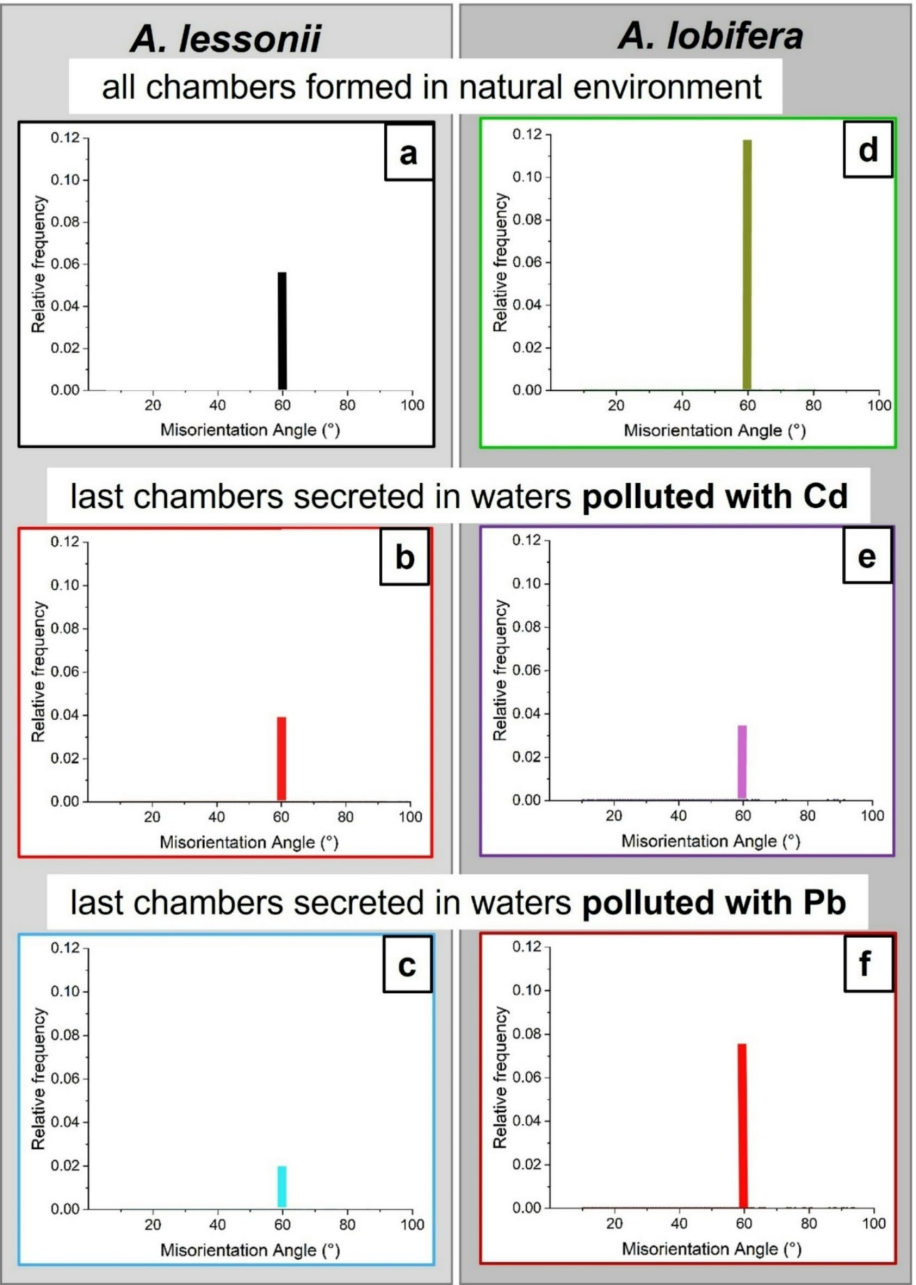
Difference in frequency of 60° misorientation for *A. lessonii* ((A) to (C)) and *A. lobifera* ((D) to (F)) chamber walls secreted in uncontaminated ((A), (D)) and contaminated ((B), (C), (E), (F)) environments. We observe: (i) a metabolic effect, with a difference in the height of the 60° misorientation peak for the shells of the two investigated species and (ii) a “polluting agent” effect, with a difference for the height of the 60° misorientation peak, thus twin formation, for Cd^2+^ and Pb^2+^.

 EBSD measurements allow the measurement of misorientation between crystals and between twin domains. We show misorientation with relative frequency – misorientation angle diagrams (Figs. 7, 8, 9, 10, 11 and 12). If one observes for an EBSD scan a peak at a specific twin-related misorientation (e.g. for calcite, for the 60°-(001) twin a marked peak at 60° misorientation), then, this can be taken as an indication for formation of 60°-(001) twins in the shell section that was scanned with EBSD. Further proofs for the presence of crystal twins is given with pole figures and misorientation-distance diagrams (e.g. Figures 5 and 10 in Lastam et al. (2022)^2^, Fig. 7 in Lastam et al. (2023)^3^), information gained from EBSD measurements.

### What is the advantage of twinned crystals?

It is evident from our study that crystal twinning is used by the foraminifera studied for shell formation, even under high-stress conditions such as water contamination. Even though not yet investigated for foraminifera shells, it has been shown for man-made materials that the formation of twinned crystals initiates an increase in the strength of the stuctural material.

The properties of materials, man-made or biologically secreted, can be changed in several ways: by incorporating impurities into crystals (e.g. Mg, organic substance), by crystal organization patterns (microstructure), by forming composites of minerals or by forming composites of a mineral/minerals and organic substance^47^. Nonetheless, work of the last decades highlights a further way of improving material properties of a structural hard tissue through the incorporation of boundaries and interfaces into the crystal lattice^47,48,51,52^. This mode of material property improvement is advantageous because it does not alter the chemical composition. For man-made hard tissues it is well known that grain boundaries act as obstacles against dislocation motion^51,52^. This causes strengthening and is known as the Hall–Petch hardening effect^47,48^. Accordingly, the strength of a structural hard tissue increases with decreasing grain/crystal size and an increase in grain boundaries.

The interfaces between twin domains are also boundaries. They are, however, special boundaries. Twin boundaries are low-energy, highly coherent and stable boundaries. For man-made materials, twin boundaries are straight. For biologically-formed hard tissues, twin boundaries are undulated (e.g^2,3,6,31^). The latter results from the curved/undulated morphology of the biocrystals (e.g. Figure 9g in Yin et al. (2021)^6^). Hence, there is a marked difference in twin boundary architecture between man-made and biologically secreted structural materials. As twin boundaries are crystal orientation- and microstructure-dependent^53–55^, they are distributed in the hard tissue in 3D and form, as grain boundaries do, efficient barriers to dislocation motion, block crack propagation, increase fracture toughness and stabilize the nanostructure of the material^32,33,47,56–59^.

The effect of twin boundaries on fracture toughness is further increased by: (i) a decrease of the spacing between twin boundaries, thus, an increase in the number of twin boundaries in the crystal or (ii) generation of a hierarchical 3D twin boundary network in the structural material^53,55^. For carbonate biological structural materials we find a hierarchically organized 3D network of twin boundaries in, for example, rotaliid calcite and bivalve cross-lamellar and complex cross-lamellar aragonite. As shown by Lastam et al. (2022, 2023)^2,3^and Yin et al. (2021)^6^, the calcite that forms the foraminiferal shell wall between the primary organic sheet (POS: the template for calcite nucleation) and distal outer shell surface consists, at the start of crystal growth, of nanometer-sized fibrils. These aggregate to fibril clusters and form sheaf-shaped crystals. An array of the latter seams the surface of the distal shell (Fig. 4 in Yin et al. (2021)^6^, Fig. 1 in Sancho Vaquer et al. (2024)^4^). Twin generation in rotaliid calcite starts with the formation of fibril clusters and of the sheaf-shaped crystals, right at the POS (Fig. 10a in Lastam et al. (2023)^3^, Fig. 16 A in Sancho Vaquer et al. (2024)^4^) and is kept up to the distal, outer, shell surface. Accordingly, the distal shell portion is formed of very many twinned crystals and comprises an extensive 3D, hierarchically-organized, network of twin boundaries. Yin et al. (2021)^6^and Lastam et al. (2022)^2^investigated twin formation for a wide range of benthic and planktonic rotaliid species and, based on EBSD measurements, showed that twinned calcite is secreted by all investigated species (Figs. 15 and 16 in Lastam et al. (2022)^2^). For some species, e.g. *G. sacculifer*, *P. obliquiloculata* and *O. universa*, crystal twin formation and 3D twin boundary generation is extensive, almost the entire shell calcite consists of twinned crystals. Shell generation of twinned aragonite and incorporation of a 3D network of twin boundaries into the hard tissue is also observed for bivalves, for species that form their shell of cross-lamellar and complex cross-lamellar aragonite^31,60^. The latter microstructure is, today, the dominating microstructure of bivalve shells.

In essence, it is demonstrated by now that twin formation and twin boundary incorporation into the hard tissue improves its material properties, for biological carbonates as well as for man-made materials. The twin-initiated toughening effect is enhanced even further when the 3D network of twin boundaries is hierarchical. Although present, it is clearly shown that the twinning signal decreases in the areas grown under poisoned condition.

### The use of the crystal twin signal of rotaliida as an environmental indicator

The aim of our study is to report a further use of growth twin formation, in addition to the utilization of crystal twinning as a means of toughening the calcite. We show, subsequently, that the twin signal of modern *A. lessonii* and *A. lobifera* shells can be developed as a proxy for determination of contamination of seawater with Pb^2+^ and/or Cd^2+^.

*Growth twin formation for biological aragonites* has been often reported by now. It is reported for bivalve and gastropod shells, bivalve myostraca, polyplacophora plates and spines, coral skeletal elements and fish otoliths^11–17,31,60–67^. In contrast, even though the microstructures of biological calcites are by now well investigated, there are only few reports of *growth twin formation in calcitic shells and skeletal elements*: Floquet and Vielzeuf (2011)^68^ observed that the calcitic sclerites of the red coral *Coralium rubrum* (Linnaeus, 1758) are twinned and Castillo Alvarez et al. (2023)^69^ report the presence of nano- and micro twins in the calcite prisms of the pearl oyster *Pinctada margaritifera* (Linnaeus, 1758).

The most outstanding examples for biologically formed twinned calcite is given with the shells of benthic and planktonic Rotaliida and benthic Robertina (e.g. *A. lessonii*, *A. lobifera*, *A. tepida*, *O. universa*, *G. sacculifer*, *P. obliquiloculata*, *A. convexa*, *O. ammonoides*, *C. nitida*, *Globigerinoides conglobatus* (Brady, 1879), *Globorotalia menardii* (d’Orbigny in Parker, Jones & Brady, 1865), *Globorotalia truncatulinoides* (d’Orbigny, 1839), *Turborotalia humilis* (Brady, 1884), *Hastigerina pelagica* (d’Orbigny, 1839), *Globigerinoides ruber ruber* (d’Orbigny, 1839), *Globorotalia scitula* (Brady, 1882), *Globigerina bulloides* (d’Orbigny, 1826), *Tenuitellita fleischeri* (Li, 1987), *Neogloboquadrina dutertrei* (d’Orbigny, 1939) and *H. elegans*). The latter studies showed that the growth twin signal is not only an inherent but, also, a marked material characteristic for calcitic as well as aragonitic, for benthic as well as planktonic, Rotaliida and Robertina. Of the five, so far, described twin laws for calcite, twinning in modern foraminifera follows the 60°-(001) twin law for calcite and, for aragonite, the 63.8°-(110) twin law^6^.

#### The selective influence of *A. lessonii* and *A. lobifera* on the intensity of twin generation

We observe differences in the extent of the twin signal (this study, Sancho Vaquer (2024)^4^, Lastam et al. (2022)^2^). Lastam et al. (2022)^2^showed for the investigated Rotaliida that the extent of the 60° peak varies between (i) clade 1 and clade 2 species (Fig. 13 in Lastam et al. (2022)^2^) and that it varies between benthic and planktonic Rotaliida (Figs. 15 and 16 in Lastam et al. (2022)^2^). The authors observed that most twinned is the calcite of planktonic Rotaliida, relative to the calcite of benthic Rotaliida. Furthermore, most twinned is the calcite of those species that form chambers with a strongly spherical shape (Fig. 15 in Lastam et al. (2022)^2^), while for the calcite of species that have flattened chamber morphologies (e.g. *A. tepida*, *O. ammonoides*, Fig. 16 in Lastam et al. (2022)^2^), twinned calcite is still observed in the shell but there is also a large amount of untwinned, misoriented calcite crystallites.

Figures 9A, 10A, 11A and D and 12A and D show in the relative frequency-misorientation angle diagrams the 60°-misorientation peak for *A. lessonii* and *A. lobifera* unpoisoned, reference, shells. We find a significant difference in the height of the 60° misorientation peak (Fig. 12A, D). For *A. lessonii*, the maximal relative frequency value for the 60° misorientation peak is 0.06, for *A. lobifera* it is 0.12. Nonetheless, even though the extent of the twin signal differs between *A. lessonii* and *A. lobifera*, when shell wall secretion takes place in Cd^2+^- and Pb^2+^-poisoned water, the 60° misorientation, the twin signal, decreases markedly for both species (Figs. 9 and 10).

When exposed to poisoned water, *A. lessonii* and *A. lobifera* carry on secreting shell calcite. In comparison to the reference, this calcite is indistinguishable for most parameters of crystallographic organization except for the decrease of twin formation.

#### The selective influence Cd^2+^ and Pb^2+^ on calcite twin formation

Cd^2+^ and Pb^2+^both substitute into the calcite octahedral sites^70,71^, although this is preferred for Cd^2+^, which is incorporated deep into the material, while for Pb^2+^, it mostly is incorporated onto the surface of the calcite crystal. The substitution of Cd^2+^ and Pb^2+^ions is a very favourable exhothermic process, and as such, can stabilise the calcite structure^72–74^.

When based on the development of the 60° misorientation peak, we find that for *A. lessonii* Pb^2+^ exerts a stronger effect, relative to the effect of Pb^2+^ for *A. lobifera* (compare Figs. 11C and 12C with Figs. 11F and 12F). This might be related to the different concentrations of Pb^2+^ in the culture conditions of the two species. Furthermore, our studies show that *A. lobifera* Cd^2+^ is the slightly stronger determinant for a change of the twin signal (compare Figs. 11E and 12E with Figs. 11B and 12B).

We observe the following trends:

1. Relative to the reference shell that was secreted in unpoisoned water, we find for both species a decrease in the twin signal of the calcite that was secreted in Cd^2+^- and/or Pb^2+^-poisoned environments.
2. The species are differently affected by Cd^2+^- and Pb^2+^-poisoning: (a) Twin formation in *A. lessonii* shells is strongly affected by addition of Pb^2+^ to the water. (b) Twin formation in *A. lobifera* shells is only slightly affected by addition of Cd^2+^ to the water.

In essence, we find for foraminiferal calcite twin formation (i) a metabolic as well as (ii) a chemical element influence. The microstructure and texture of the shells are not markedly influenced by contamination of the water with metal cations. Accordingly, shell microstructure and texture cannot be used as a proxy for identification of pollution of marine environments with Cd^2+^ and/or Pb^2+^, but the 60°-misorientation signal, the crystal twin signal, coupled to the degree of crystal twin formation.

A comparable study has been carried out with the bivalve *Mytilus galloprovincialis* (Lamarck, 1819). Hahn et al. (2012)^38^ report structural change of shell calcite of *M. galloprovincialis* specimens living in natural and in CO_2_-poisoned habitats. The experiments were carried out in the Mediterranean Sea^38^, as well as under laboratory conditions^75^. In both cases, Hahn and co-authors demonstrate that the shells record environmental change and toxification of the water with CO_2_ with calcite microstructure, texture and carbon and oxygen isotope geochemistry. Calcite twin formation did not take place, neither when secreted at unpoisoned conditions, nor when secreted in CO_2_-poisoned environments.

## Conclusions

The Ca-carbonate material of benthic and planktonic Rotaliida and benthic Robertina shells shows a specific structural characteristic: *the crystals are twinned*. Twinned carbonate formation enhances the durability and hardness of the structural hard tissue. In the present contribution, we investigate whether the “twin-related signal” of foraminiferal shell calcite is influenced and changed by environmental pollution and show that, for the investigated species, the “crystal twin-signal” can be developed as a tool for indication of Cd^2+^- and Pb^2+^-contamination of marine water.

We characterize the calcite of *A. lessonii* and *A. lobifera*, secreted in uncontaminated as well as in Cd^2+^- and Pb^2+^-contaminated water with EBSD and FE-SEM imaging. We draw the following conclusions from our results:

1. There is a marked difference between the investigated specimens of *A. lessonii* and *A. lobifera* in the extent of crystal twin formation for shell calcite secreted in uncontaminated environments.
2. Irrespective of this difference, when chamber calcite is secreted in Cd^2+^- and Pb^2+^-contaminated water, we observe a marked decrease in the 60°-misorientation twin signal for both species.
3. We do not measure a difference in shell microstructure and texture for shell sections secreted in unpolluted and in Cd^2+^- and Pb^2+^-polluted environments.
4. Cd^2+^ and Pb^2+^ exert different influence on crystal twin formation or suppression of crystal twin formation.
5. *A. lessonii* and *A. lobifera* react differently to Cd^2+^ and Pb^2+^ when secreting twinned calcite.
6. This study focuses on heavy metal pollution at very high concentrations. In the case of Cd^2+^, the concentration is 165–166 µg L^−1^. Cadmium incorporation is also used as a paleonutrient proxy. In these investigations, the concentrations are much lower, up to 0.135 µg L^−1^, as noted by Boyle (1988)^76^and Bryan and Marchitto (2010)^77^. We have observed alterations in the twinning signal under the aforementioned very high concentrations. However, further research is necessary in order to fully comprehend how these results can be translated to natural environments, in particular to determine nutrient quantities. Considering our findings, we propose developing the twinning signal as a possible environmental and paleonutrient proxy through the further analysis of twinning signals of modern specimens across a wide spectrum of pollutant concentrations and nutrient levels^22^.

## Electronic supplementary material

Below is the link to the electronic supplementary material.

Supplementary Material 1

## Data Availability

The datasets generated during and/or analysed during the current study are available from the corresponding author on reasonable request.
