# Supplementary material for: Amphistegina lessonii and Amphistegina lobifera shell microstructure, texture and twinning pattern reflect resilience to cadmium and lead
Source: Sci Rep. 2025 Apr 26;15:14617. doi: 10.1038/s41598-025-94811-7 (PMC12033310; doi:10.1038/s41598-025-94811-7)
Supplement: Supplementary file 1 — Supplementary Material 1 [file 41598_2025_94811_MOESM1_ESM.doc]

**Supplementary Information**

**
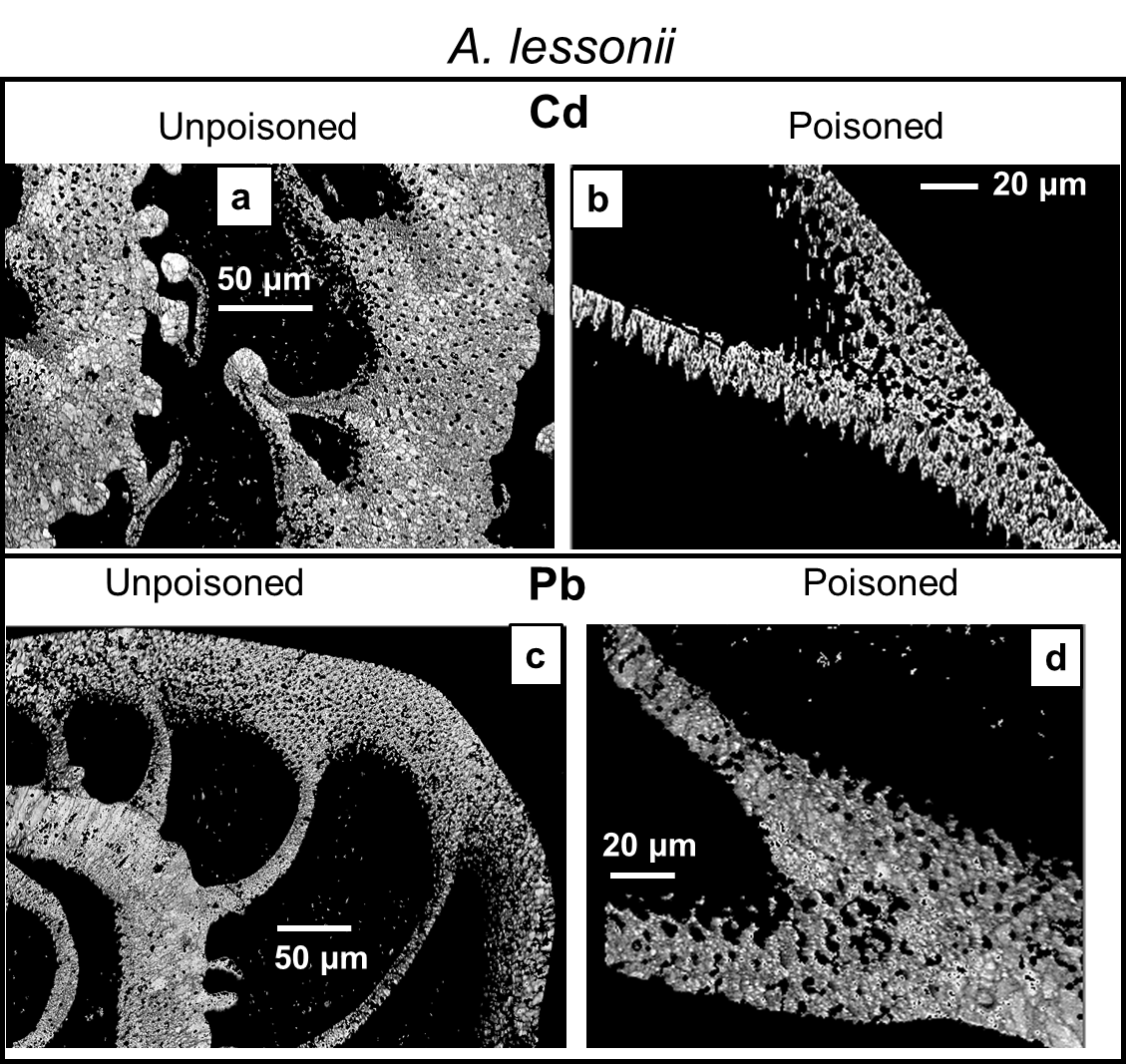
**

**Figure S1.** Band contrast measurements of *A. lessonii* specimens that lived first in natural environments ((A), (C)) and, for the secretion of the last few chambers, lived in water contaminated with either Cd^2+^ or Pb^2+^ ((B), (D)). In those shells where calcite growth takes place entirely in natural environments, the morphology of crystals within the at last formed chambers is euhedral and crystal sizes are in the very few micrometer range (this study and Fig. 11 in Yin et al. (2021)[6]). This is also the case at last secreted chamber walls of species that were secreted in polluted environments ((B), (D)). There is no marked difference in microstructure, crystal size or crystal morphology between shell sections secreted under natural and under poisoned conditions.

**
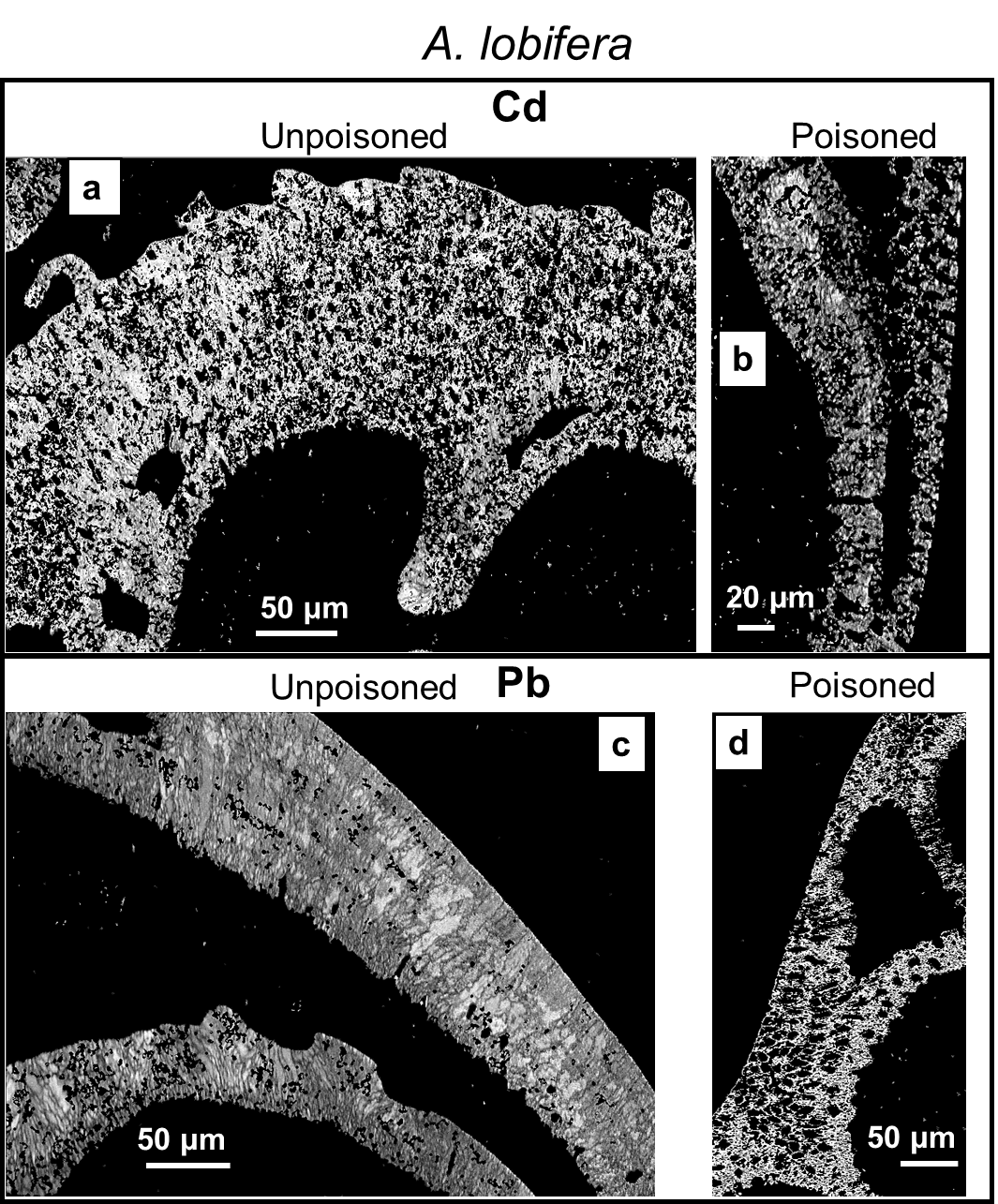
**

**Figure S2.** Band contrast measurements of *A. lobifera* specimens that lived first in natural environments ((A), (C)) and, for the secretion of the last few chambers, were transferred into water contaminated with either Cd^2+^ or Pb^2+^ ((B), (D)). There is no marked difference in microstructure between shell sections secreted under natural and under Cd^2+^- or Pb^2+^-poisoned conditions.


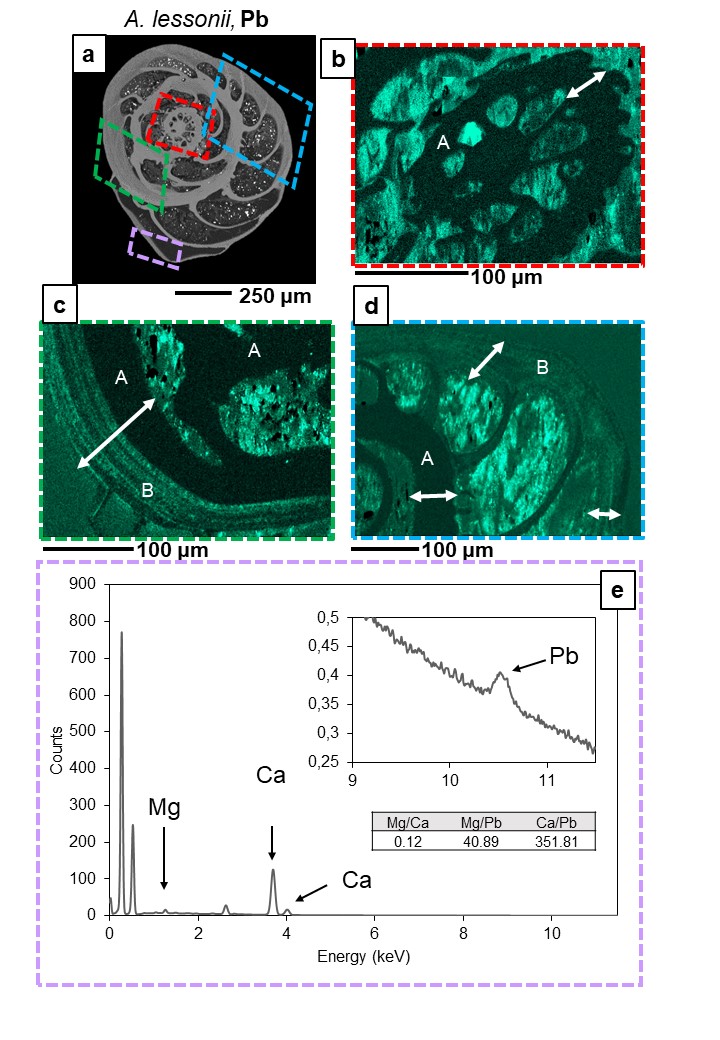


**Figure S3.** (a) Cut surface of A. lessonii poisoned by Pb^2+^, with the areas analyzed (red, green, blue and purple) for EDS.(b-d) Element distribution maps of the analyzed areas for Pb^2+^, corresponding to each color. White arrows indicate the shell width. Unpoisoned shell sections are indicated by A. Shell sections grown under poisoned conditions are indicated by B. The red area (b) shows an unpoisoned shell wall, while (c) and (d), in green and blue respectively, show Pb^2+^ poisoning. A clear zonation can be seen in both, and in (c), the difference between poisoned and unpoisoned shell wall areas can be observed for the same wall. (e) EDS spectra map showing the overall spectra and a zoomed-in insert illustrating the Pb^2+^ peak. The full EDS spectra of the measurement site (purple) and a zoomed-in insert illustrating the Pb^2+^ peak are displayed. Ca and Mg peaks are also indicated, with the ratios of peak heights calculated and shown.

**
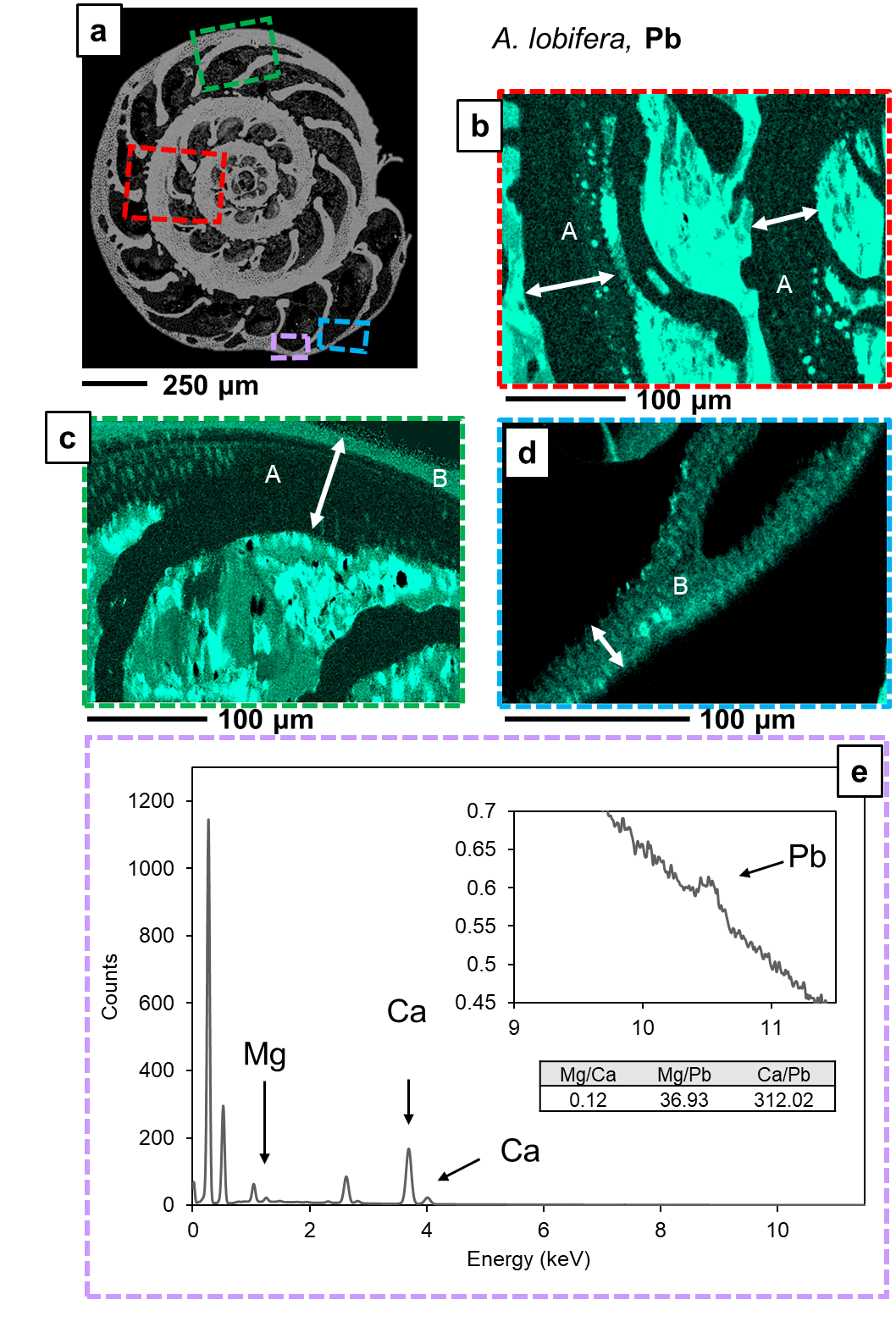
**

**Figure S4.** (a) Cut surface of A. lobifera poisoned by Pb^2+^, with the areas analyzed (red, green, blue and purple) for EDS.(b-d) Element distribution maps of the analyzed areas for Pb^2+^, corresponding to each color. White arrows indicate the shell width. Unpoisoned shell sections are indicated by A. Shell sections grown under poisoned conditions are indicated by B. The red area (b) shows an unpoisoned shell wall, while (c) and (d), in green and blue respectively, show Pb^2+^ poisoning. In (c), the difference between poisoned and unpoisoned shell wall areas can be observed for the same wall. (e) EDS spectra map showing the overall spectra and a zoomed-in insert illustrating the Pb^2+^ peak. The full EDS spectra of the measurement site (purple) and a zoomed-in insert illustrating the Pb^2+^ peak are displayed. Ca and Mg peaks are also indicated, with the ratios of peak heights calculated and shown.
